# The Josephin domain (JD) containing proteins are predicted to bind to the same interactors: Implications for spinocerebellar ataxia type 3 (SCA3) studies using *Drosophila melanogaster* mutants

**DOI:** 10.3389/fnmol.2023.1140719

**Published:** 2023-03-15

**Authors:** Rita Sousa e Silva, André Dias Sousa, Jorge Vieira, Cristina P. Vieira

**Affiliations:** ^1^Instituto de Investigação e Inovação em Saúde (i3S), Universidade do Porto, Porto, Portugal; ^2^Instituto de Biologia Molecular e Celular (IBMC), Universidade do Porto, Porto, Portugal

**Keywords:** ataxin-3, Josephin domain, polyQ, SCA3, *Drosophila*, protein–protein interaction

## Abstract

Spinocerebellar ataxia type 3, also known as Machado-Joseph disease (SCA3/ MJD), is the most frequent polyglutamine (polyQ) neurodegenerative disorder. It is caused by a pathogenic expansion of the polyQ tract, located at the C-terminal region of the protein encoded by the *ATXN3* gene. This gene codes for a deubiquitinating enzyme (DUB) that belongs to a gene family, that in humans is composed by three more genes (*ATXN3L*, *JOSD1*, and *JOSD2*), that define two gene lineages (the *ATXN3* and the Josephins). These proteins have in common the N-terminal catalytic domain (Josephin domain, JD), that in Josephins is the only domain present. In *ATXN3* knock-out mouse and nematode models, the SCA3 neurodegeneration phenotype is not, however, reproduced, suggesting that in the genome of these species there are other genes that are able to compensate for the lack of *ATXN3*. Moreover, in mutant *Drosophila melanogaster*, where the only JD protein is coded by a Josephin-like gene, expression of the expanded human *ATXN3* gene reproduces multiple aspects of the SCA3 phenotype, in contrast with the results of the expression of the wild type human form. In order to explain these findings, phylogenetic, as well as, protein–protein docking inferences are here performed. Here we show multiple losses of JD containing genes across the animal kingdom, suggesting partial functional redundancy of these genes. Accordingly, we predict that the JD is essential for binding with ataxin-3 and proteins of the Josephin lineages, and that *D. melanogaster* mutants are a good model of SCA3 despite the absence of a gene from the *ATXN3* lineage. The molecular recognition regions of the ataxin-3 binding and those predicted for the Josephins are, however, different. We also report different binding regions between the two ataxin-3 forms (wild-type (wt) and expanded (exp)). The interactors that show an increase in the interaction strength with exp ataxin-3, are enriched in extrinsic components of mitochondrial outer membrane and endoplasmatic reticulum membrane. On the other hand, the group of interactors that show a decrease in the interaction strength with exp ataxin-3 is significantly enriched in extrinsic component of cytoplasm.

## 1. Introduction

Although polyglutamine repeats (polyQ) are the most abundant homorepeats in eukaryotic proteomes (Mier and Andrade-Navarro, 2021), they are frequently associated with debilitating and incurable neurodegenerative disorders, known as polyQ diseases (Heemels, 2016; Gitler et al., 2017). PolyQ diseases, are a family of nine rare dominantly transmitted disorders caused by a pathogenic expansion of CAG (Cytosine-Adenosine-Guanine) trinucleotide repeats in the corresponding coding region of the nine genes (Androgen receptor, Atrophin 1, Ataxin- 1, 2, 3, 7, calcium voltage-gated channel subunit alpha 1A, Huntingtin, and TATA-binding protein). Although these genes are unrelated, polyQ diseases share resemblances, including the mutational mechanism (with a negative correlation between the length of CAG repeats and age of onset, and positive correlation with the disease severity) and a similar phenotype with progressive neurodegeneration due to aggregates formation (Golding, 1999; Huntley and Golding, 2000; Huntley and Clark, 2007). One of these diseases, with high prevalence worldwide (1:50,000–100,000 individuals), is spinocerebellar ataxia type 3/Machado-Joseph disease (SCA3/MJD), also known as Azorean disease of nervous system, due to the highest predominance in Azores population (Bettencourt et al., 2010a; Raposo et al., 2017). It is caused by a pathogenic expression of the polyQ tract of the ataxin-3 protein, encoded by the *ATXN3* gene (Matos et al., 2019; McLoughlin et al., 2020).

Ataxin-3 is 361 amino acids long and is constituted by a N-terminal Josephin domain (JD, residues 1–180), a C-terminal unstructured tail with a polyQ tract (residues 292–305), and three ubiquitin interaction motifs (UIMs, residues 224–243, 244–263, and 331–349; Deriu et al., 2014). Although different isoforms exist due to alternative splicing, mostly at the C-terminal region, this is the most common form in the brain (Bettencourt et al., 2010b). The presence of a JD and the UIMs imply a ubiquitination-proteasome regulation function for this protein (Mao et al., 2005). Indeed, ataxin-3 belongs to the Josephin family of deubiquitination enzymes superfamily (DUB), a class of proteins crucial for efficient removal of ubiquitin from its conjugates (Burnett et al., 2003; Winborn et al., 2008; Vlasschaert et al., 2017). *Via* DUB activity, ataxin-3 interacts with molecular chaperones and proteins of the ubiquitin-protease system such as E3 ubiquitin protein ligases (70 kDa heat shock protein interacting protein CHIP (Jana et al., 2005), gp78 (Ying et al., 2009), E4B (Matsumoto et al., 2004), HRD1 (Wang et al., 2006), Parkin (Tsai et al., 2003; Durcan et al., 2011), and MITOL (Sugiura et al., 2011)), tightly regulating its activity, and thus, maintaining normal cellular homeostasis (Ciechanover and Brundin, 2003). Ataxin-3 also interacts with proteins involved in proteasomal degradation, by clearance of misfolded proteins *via* endoplasmatic reticulum associated degradation, such as valosin-containing protein VCP/p97, Rad23, hHR23A and hHR23B (Wang et al., 2000; Zhong and Pittman, 2006). Based on the interaction with transcriptional activators, such as CREB-binding protein (CBP), p300, p300/CBP-associated factor, and histone deacetylase 3 (HDAC3), ataxin-3 is also involved in transcription regulation (Li et al., 2002; Evert et al., 2006; Rodrigues et al., 2007; Chou et al., 2008). Ataxin-3 has been also described to be involved in the maintenance of genome integrity, since it interacts with polynucleotide kinase 3′-phosphatase (Chatterjee et al., 2015; Gao et al., 2015) and checkpoint kinase 1 (Tu et al., 2017). For a review of the multiple functions of ataxin-3, see for instance, Matos et al. (2019).

In humans, there are three other deubiquitinating enzymes, other than ataxin-3, that present a JD, namely ataxin-3-like (ataxin3L), Josephin-1 (JOS1), and Josephin-2 (JOS2; Tzvetkov and Breuer, 2007; Weeks et al., 2011). These proteins are subdivided in two subgroups, the ataxins (ataxin-3/ataxin3L) and the Josephins (JOS1/JOS2; Tzvetkov and Breuer, 2007). In humans the genes encoding Josephins show expression patterns similar to *ATXN3* in areas of neuronal degeneration in SCA3 patients (the subthalamopallidal, dentatorubral, pontocerebellar, and spinocerebellar systems, and lower motoneurons; Nishiyama et al., 1996), in contrast with *ATX3L*, that is not expressed in brain. This family of proteins is conserved in eukaryotes, especially in the Metazoans, including invertebrates and vertebrates (Tzvetkov and Breuer, 2007; Sowa et al., 2009; Weeks et al., 2011), although there are differences in gene number and also in gene lineage presence. For instance, in *Drosophila* there is only one DUB gene, from the Josephin lineage (*CG3781*, encoding for Josephin-like protein, JosL). In Catarrhini species (Cercopithecidae, Hominidae and Hylobidae) there is a *ATXN3* duplication, that originate the *ATXN3L* gene (Weeks et al., 2011). Because of the high structural similarity of Josephins, in particular JOS1, with ataxin-3 in their catalytic domain, it has been hypothesized that their functions might be regulated by ataxin-3, and thus could be involved in SCA3 (Seki et al., 2013). Indeed, mice and nematodes lacking *ATXN3* showed no neurodegeneration phenotype, also suggesting functional redundancy (Mao et al., 2005; Rodrigues et al., 2007; Schmitt et al., 2007). Nevertheless, the number of species analyzed so far is small, and given the possible redundancy of Josephins and *ATXN3*, it is conceivable that significant differences exist between species in the number of deubiquitinating enzymes. Therefore, here we address the evolution of this gene family, by performing a phylogenetic analysis using 756 annotated RefSeq animal genomes available at NCBI.

Mutant flies, expressing the expanded human *ATXN3*, unveiled key pathways and validated the toxicity of several protein interactions in SCA3 disorder (Bilen and Bonini, 2007; Alves et al., 2010; Zhang et al., 2010; Vobfeldt et al., 2012; Xu et al., 2015; Bonini, 2022). Indeed, when using the human *ATXN3* expanded form (coding for a protein with 78Q) to screen for modifiers of polyQ toxicity in *Drosophila*, these were found to be enriched in ubiquitination-proteasome components, protein turnover/quality control, transporters or transcription regulation components (Vobfeldt et al., 2012). Several protein–protein interactions (PPIs) and their contribution to the disease were also identified in other *Drosophila* screens, with misexpression screens for modifiers of polyQ toxicity (Bilen and Bonini, 2007) and RNAi screens for modifiers of polyQ aggregation (Zhang et al., 2010). It should be noted that this data is not available in the main PPI databases for humans, since these are interactions between proteins from different species, and thus it is unclear whether the interactions of the homologous proteins are present in humans. Since there is no *ATXN3* lineage gene in *Drosophila,* and there is only one Josephin-like gene (Weeks et al., 2011), here we address whether mutant *ATXN3* flies are a good model for SCA3, by comparing the ataxin-3 interaction regions of human and fly homologous proteins, using the *in-silico* approach of Rocha et al. (2019).

The regions of ataxin-3 interaction vary, and for ubiquitination-proteasome proteome NEDD8, Parkin (PRKN), and CHIP, the interactions are mostly at the ataxin-3 JD and the UIM domains (see Figure 1 in Matos et al., 2019). The UIM domains are crucial for the binding of cAMP-response-element binding protein (CBP), Lysine Acetyltransferase 2B (KAT2B/PCAF) and histone acetyltransferase p300 (P300). The C-terminal region, in particular the NLS site, is however, essential for VCP/p97 interaction (Fujita et al., 2013; Matos et al., 2019). The relative frequency of use of the different ataxin-3 interacting regions is, however, unknown, and here we address this issue by performing *in-silico* protein–protein docking analyses (Rocha et al., 2019) using 150 ataxin-3 interactors.

**Figure 1:**
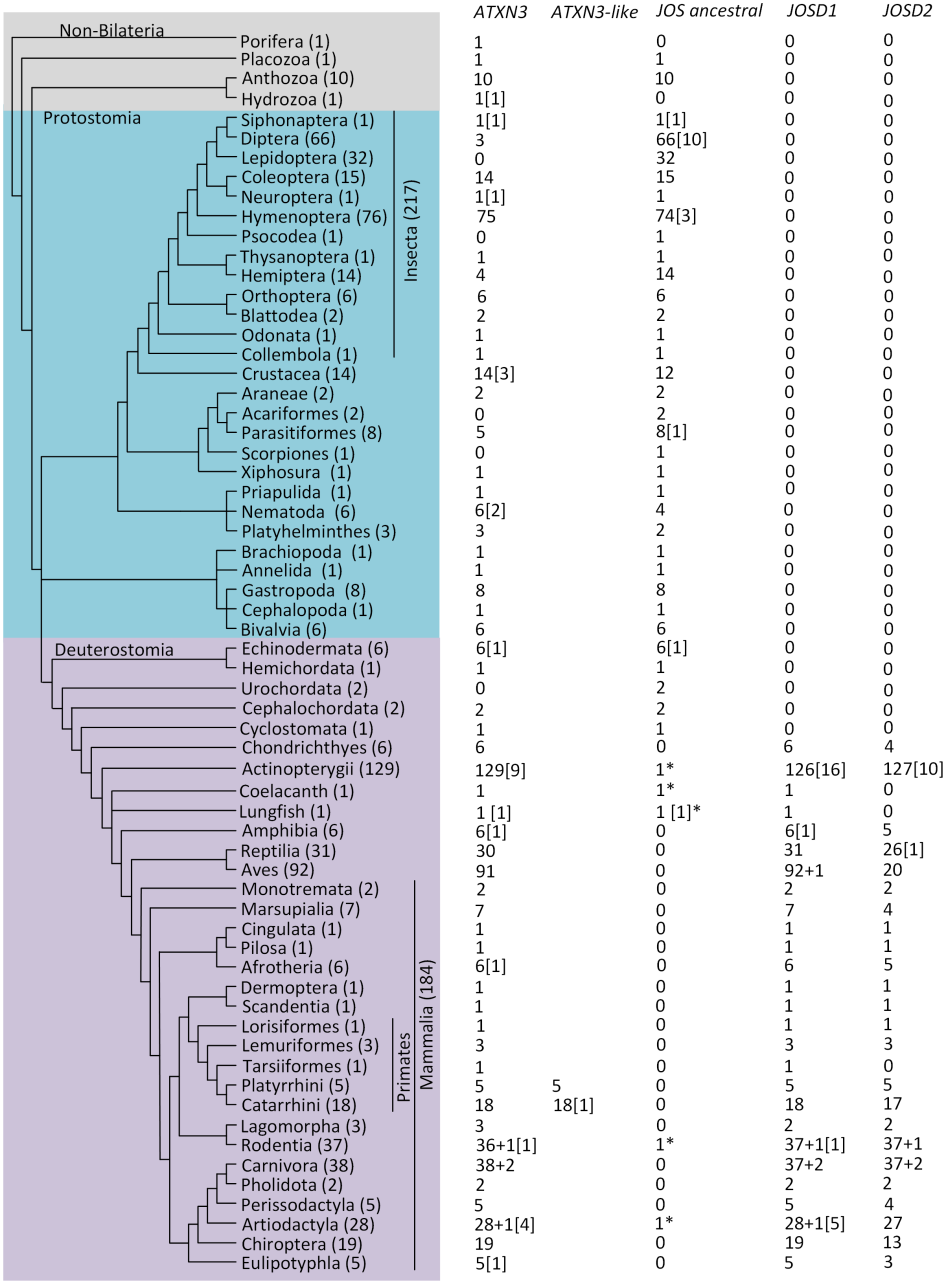
The ataxin-3 and Josephin lineages evolution across the animal kingdom, based on 747 species. The cladogram topology is depicted as in the Tree of Life web project (Maddison et al. 2007). For each taxomomic group, the number of genomes analyses is indicated within brackets after the taxonomic group. Numbers under the gene names indicate the number of genomes where the gene has been identified. Gene duplications are indicated within square brackets. Stars represent likely phylogenetic inferences errors.

PolyQ regions are located between a disordered region and a coiled-coil region used for PPI (Schaefer et al., 2012; Mier and Andrade-Navarro, 2021), and when expanded, this region is hypothesized to adopt a helical conformation, extending the preceding helix, and thus making the PPI stronger (Schaefer et al., 2012; Petrakis et al., 2013; Mier and Andrade-Navarro, 2021). These structural changes of the protein (Takeuchi and Nagai, 2017) lead to different accessibility at specific interacting residues that are needed for the normal protein activity (Lim et al., 2006; Araujo et al., 2011; Suter et al., 2013; Hosp et al., 2015; Silva et al., 2018; Rocha et al., 2019). To address this issue comparative *in-silico* protein–protein docking analyses have been performed, using the pathogenic (exp) and the wild type (wt) ataxin-3 forms. The identification of the proteins that interact differently with the two ataxin-3 forms, can lead to novel therapeutic approaches.

## 2. Materials and methods

### 2.1. Phylogenetic analyses

Coding sequences (CDS) of 756 annotated RefSeq Animal genomes, corresponding to 751 species (there are 3, 2, 2, and 2 annotated genomes for *Canis lupus*, *Cricetelus griseus*, *Gallus gallus,* and *Bos indicus*, respectively) were downloaded from NCBI Assembly database (October 2022). Using SEDA (López-Fernández et al., 2019, 2022); a Docker image is available at the pegi3S Bioinformatics Docker Images project^1^ (López-Fernández et al., 2022), the following operations were performed separately on each FASTA CDS file, in order to select the sequences used to perform phylogenetic analyses: (1) a tblastn search using 12 query sequences (*Homo sapiens* (P54252, Q9H3M9, Q15040, Q8TAC2); *Mus musculus* (XP_030102382.1, NP_083068.1, XP_030098747_1); *Drosophila melanogaster* (Q9W422); *Caenorhabditis elegans* (O17850, NP_871685); and *Trichoplax adhaerens* (XP_002108665, XP_002109527)). These proteins are, according to UniProt^2^ the reference isoform sequences; (2) identical CDS encoded by different transcripts were removed; (3) only sequences that are 10% larger or 10% smaller than any of the 12 sequences used as query in the tBlastn operation were kept, thus eliminating potential erroneous CDS annotations; (4) the results for each genome were merged and identical sequences removed; (5) if two sequences share an identical nucleotide region larger than 250 bp, as it is the case of CDS coding for different protein isoforms, only the sequence that is closer to the *ATXN3* size is kept; (6) headers were edited to keep only the CDS accession number; (7) information on the species, family and class was added to the sequence headers; (8) sequences showing in-frame stop codons were removed; (9) stop codons were removed; and (10) all files were merged into a single one. At the end of this protocol, 1,823 sequences remain. For four species only, no sequences remained.

The set of 1823 sequences were translated using EMBOSS transeq (Rice et al., 2000), aligned using ClustalOmega (Sievers et al., 2011), the corresponding nucleotide alignment obtained using TranslatorX (Abascal et al., 2010), and a maximum-likelihood tree obtained using Fasttree (Price et al., 2009, 2010), using standard parameters. These analyses were performed using the Docker images that are available at the pegi3S Bioinformatics Docker Images project^3^^,^^4^^,^^5^^,^^6^ (see text footnote 1; López-Fernández et al., 2022).

### 2.2. Ataxin-3, JOS1, JOS2, and JosL interactors

Ataxin-3 (Gene ID 4287), JOS1 (Gene ID 9929), JOS2 (Gene ID 126119) and JosL (Gene ID 31560) interactors were obtained from EvoPPI3^7^^,^^8^ (Vázquez et al., 2019), using the same species search, level 1 interactions (proteins that interact directly with query protein), and all databases available in this web platform. Fly genes reported as ataxin-3 modifiers (521 genes from Vobfeldt et al., 2012; Supplementary Table S1), 126 from (Zhang et al., 2010; Supplementary Table S2), and 18 genes from (Bilen and Bonini, 2007; Supplementary Table S3) were also considered in this study.

To identify the human paralogs of fly genes, we used DIOPT (DRSC Integrative Ortholog Prediction Tool^9^; Hu et al., 2011) without filters and with all the available algorithms. In the case of Vobfeldt et al. (2012) data, the results were manually verified by looking at the transformant ID provided by Vienna Drosophila Resource Centre (VDRC). When multiple fly genes were retrieved, the Ensembl (Yates et al., 2020) orthologs database was used to select those to be studied. Venn diagrams^10^ were used to identify genes that are on both the human ataxin-3 interactome and fly paralogs lists.

### 2.3. *In-silico* interaction predictions

The interaction regions between ataxin-3 and 150 selected interactors (35% of the total network; Supplementary Table S4), JosL and four fly interactors (Supplementary Table S5), JosL and 45 fly ataxin-3 modifier genes for which the human paralogs code for an ataxin-3 interactor (28% of the total network; Supplementary Table S6), JOS1 and 37 JOS1 interactors (Supplementary Table S7), JOS1 and 126 ataxin-3 interactors not reported as JOS1 interactors, JOS2 and 17 JOS2 interactors (Supplementary Table S7), and JOS2 and 129 ataxin-3 interctors not reported as JOS2 interactors were predicted using the *in-silico* methodology, described by Rocha et al. (2019).

The protein sequences of the 252 interactors analyzed were retrieved from UniProt (see text footnote 2). We used two ataxin-3 sequences, the wt form with 14Q and the exp form with 50Q. The 3D structure of these proteins was predicted using I-TASSER (Roy et al., 2010; Yang et al., 2015; Yang and Zhang, 2015). Although AlphaFold is considered the best tool for 3D structure prediction (CASP14 edition; Jumper et al., 2021), for ataxin-3 only the wt ataxin-3 struture is available, and thus, for consistency we have used I-TASSER (that in CASP7 to CASP11 was in the top ranking (Roy et al., 2010; Yang and Zhang, 2015). Due to I-TASSER protein size limitations^11^ (Roy et al., 2010; Yang et al., 2015; Yang and Zhang, 2015), five ataxin-3 interactors, longer than 1,500 residues were excluded from the analyses (Supplementary Table S4) and one fly interactor (Supplementary Table S6).

For each protein, the interacting residues (actives and passives) were predicted using CPORT^12^ (de Vries and Bonvin, 2011). For two human proteins this software was unable to produce results (Supplementary Table S4).

Docking predictions for JosL, wt and exp ataxin-3, as well as JOS1 and JOS2, with the human and/or fly interactors were obtained using HADDOCK^13^ (van Zundert et al., 2016). Only the clusters with the *z*-score ≤ 0 were used (van Zundert et al., 2016). The docking predictions failed for two human proteins (Supplementary Table S4), and one fly protein (Supplementary Table S6). The identification of the best cluster and the best structure representation of the interaction, according to the number of interfacing residues at the reference protein, was performed using PDBePISA^14^ (Krissinel and Henrick, 2007) and the Docker image pisa_xml:extract, provided at pegi3S under utilities^15^ (see text footnote 1; López-Fernández et al., 2021). In case of a tie we use the structure representation of the interaction with the highest number of interactions. If the tie still holds, we use the solvent-accessible area (Å) interface to choose the best cluster and structure representation of the interactor. Furthermore, since interactors that show six or more interactions at the polyQ tract have been associated with low confidence predictions (Rocha et al., 2019), in order to be conservative, these were excluded from the analyses (Supplementary Table S8). It should be noted that, the mechanisms by which polyQ modulate PPI might be through the expansion of sequence-adjacent coiled-coil regions that facilitates the interaction with a coiled-coil region from another protein (Mier and Andrade-Navarro, 2021). For wt and exp ataxin-3 and JosL, interaction regions were identified by looking for amino acid positions that interact with more than 50% of the interactors and that are less than three consecutive amino acids apart from each other.

The protein structure was visualized with PyMol (The PyMOL Molecular Graphics System, Version 1.7.4 Schrödinger, LLC), as a docker image available at pegi3S^16^ (see text footnote 1; López-Fernández et al., 2021).

### 2.4. Interactor’s characterization

The selected human ataxin-3 interactors here studied are not enriched in a particular molecular function when performing Gene Ontology enrichment analysis (ATP-dependent activity (10), binding (59), catalytic activity (54), cytoskeletal motor activity (2), molecular function regulator (9), molecular transducer activity (1), structural molecule activity (3), transcription regulator activity (6), translation regulator activity (1), transporter activity (6), no PANTHER category assigned (34)) using PANTHER (Ashburner et al., 2000; Mi et al., 2019; Gene Ontology, 2021).

Human interactors tissue expression was addressed using the spatial profiling of the human Brain, according to The Human Protein Atlas^17^, for the regions that are relevant for SCA3 (Seidel et al., 2012), namely the basal ganglia, cerebral cortex, midbrain, thalamus, pons and medulla oblongata. For *Drosophila* interactors, tissue expression was verified in FlyAtlas 2: the *Drosophila* expression atlas^18^ (Leader et al., 2018).

### 2.5. Statistical analysis

Statistical analyses were performed using SPSS, version 27.0 (IBM Corp. Released, 2020) using non-parametric tests. If the data is paired, we used a Sign test, while if the two samples are independent, a Mann–Whitney U test was used. The significance level is 5%.

## 3. Results

### 3.1. Ataxin-3 family evolution in animals

The summary of the phylogenetic analyses performed using 1823 sequences from 747 species (see section “Materials and Methods” and Supplementary Figures S1A,B) is shown in Figure 1 after this region. Both the *ATXN3* and the ancestral *JOSD* genes can be found in Non-bilateria animals. Therefore, these genes belong to two old gene lineages. Nevertheless, in Porifera, that are arguably the earliest-branching metazoan taxon (Wörheide et al., 2012), only the *ATXN3* gene was found. This could suggest that the ancestral *JOSD* gene lineage arose by duplication of the *ATXN3* gene after the separation of sponges from the other non-bilateria groups. Nevertheless, only a single Porifera genome was analyzed, and thus one cannot be confident that the ancestral JOS gene is missing in Porifera. Moreover, given that genes belonging to the *ATXN3* and *JOSD* lineage have been described in non-animal species (Scheel et al., 2003), such interpretation cannot be made at present with confidence.

For Prostotomia, with the clear exception of Diptera and Lepidoptera, one *ATXN3* and one ancestral *JOSD* gene are generally found. In Psocodea, Acariformes and Scorpiones no *ATXN3* gene was found but only one, two, and one species were analyzed, respectively. Given the low sample size, and the possibility that the genes of interest may not be annotated in the genome, no strong conclusion can be made regarding these three groups. The *ATXN3* gene can only be found in three (belonging to the Sciaridae, Cecidomyiidae, and Tephritidae families) out of 66 Diptera species. The species of the Sciaridae and Cecidomyiidae families are the only representatives of the Bibionomorpha in our dataset. Nevertheless, according to (Wiegmann et al., 2011), the Culicomorpha group branched earlier than the Bibionomorpha group, and the Culicomorpha are represented by 13 species, where no *ATXN3* gene is present, implying at least two independent *ATXN3* gene losses within Diptera. Moreover, besides *Rhagoletis zephyria* (Tephritidae) where an *ATXN3* gene sequence can be found, there are eight other Tephritidae species present in our dataset where the *ATXN3* gene was not found, including another species from the same genus (*Rhagoletis pomonella*). Therefore, within Diptera, multiple independent loss events of the *ATXN3* must have happened. In the only representative in our dataset of the Diptera sister group (Siphonaptera), the *ATXN3* gene was found. Therefore, the complete loss of the *ATXN3* gene in Lepidoptera must be another independent *ATXN3* loss event. Regarding Hemiptera, the *ATXN3* gene was found in only four out of the 14 species analyzed, while the ancestral *JOSD* sequence was found in 14 out of 14 species. This is an indication that in this group the *ATXN3* gene is also being independently lost. All insect sequences resulting from the Blast operation (first operation of the sequence retrieval protocol; see section “Materials and Methods”) were kept. Therefore, the pattern observed for insects is not due to the removal of putative *ATXN3* sequences during the sequence retrieval protocol.

Within Deuterostomia, the basal Echinodermata, Hemichordata, and Cephalochordata taxonomic groups always show a single *ATXN3* and ancestral *JOSD* gene, while Urochordata species only show a single ancestral *JOSD* gene. Although the sample size is too small to be sure (N = 2), it is possible that the *ATXN3* gene has been lost in Urochordates.

A two-round whole genome duplication (2R-WGD) event has likely occurred within the Craniata subphylum, after the separation of vertebrates from invertebrate chordates (Dehal and Boore, 2005; Van de Peer et al., 2009; Kasahara, 2013). One version of this hypothesis considers that the first round of WGD affected the common ancestor of all vertebrates, while the second affected the common ancestor of jawed vertebrates, after the separation from jawless vertebrates (Kasahara et al., 2007; Kasahara, 2013). While there is no evidence for the duplication of the *ATXN3* gene at either moment in time, the duplication of the ancestral *JOSD* gene (that originates the *JOSD1* and *JOSD2* genes) coincides with the second round of WGD (Figure 1). Indeed, in jawless vertebrates, such as the Cyclostomata, a single *JOSD* gene has been found, while in basal jawed vertebrates (Chondrichthyes) sequences representative of the *ATXN3*, *JOSD1* or *JOSD2* genes have been found. Therefore, the duplicates of the *ATXN3* and *JOSD* genes, resulting from the first round of WGD have been lost, while only the *ATXN3* duplicate, resulting from the second round of WGD has been lost.

A WGD event has been proposed in the lineage leading to teleost fish (Glasauer and Neuhauss, 2014). Nevertheless, only in between 7.0% (9/129) and 12.7% (16/126) of the Actinopterygii species show a duplication of the *ATXN3*, *JOSD1* or *JOSD2* genes (Figure 1). Therefore, the duplicates of these genes, resulting from the WGD event, have been lost. Of the nine species that show *ATXN3* gene duplications, five are from the Salmonidae and two from the Cyprinidae families. Since, a WGD has been also extrapolated within the salmonids and some cyprinids (Glasauer and Neuhauss, 2014), at least seven out of the nine *ATXN3* gene duplications can be directly associated with these events. The remaining two gene duplications are rare, independent, phylogenetically localized *ATXN3* gene duplication events, not related to WGD events. The same reasoning applies to the *JOSD1* and *JOSD2* gene duplicates. Indeed, of the 16 species that show *JOSD1* gene duplicates, 10 and 3 belong to the Salmonidae and Cyprinidae families, respectively. Moreover, of the 10 species that show *JOSD2* gene duplicates, 3 and 5 belong to the Salmonidae and Cyprinidae families, respectively. There is only one sequence coming from the species *Lates calcarifer* (family Centropomidae; Actinopterygii) that unexpectedly clusters with sequences that represent the ancestral *JOSD* gene. In this species, sequences representing the *ATXN3*, *JOSD1* or *JOSD2* genes have been found. Therefore, the misplaced sequence must be a duplicate of either *JOSD1* or *JOSD2*, although the possibility that this sequence is the result of a miss-annotation of the genome cannot be ruled out.

One sequence from both Coelacanth and Lungfish cluster with sequences representing the ancestral JOSD gene. These sequences likely represent the *JOSD2* gene, since in these two lineages, this is the only gene that was expected to be found but was not identified in the phylogenetic analyses here performed.

Within Amphibia, only *Xenopus laevis* shows a duplication of the *ATXN3* and *JOSD1* genes, which is expected, since this species is a known allotetraploid (Session et al., 2016).

Within Reptilia a single *ATXN3*, *JOSD1* and *JOSD2* gene is likely always present, although in Aves where the *ATXN3* and *JOSD1* is likely always present, only 20 out of 92 (21.7%) species show a *JOSD2* gene. There is no obvious taxonomic relationship between those species that show a *JOSD2* gene, which implies multiple independent losses of the *JOSD2* gene in Aves.

In Mammalia, with the exception of the Platyrrhini and Catarrhini primates, the rule is clearly the presence of a single copy for *ATXN3*, *JOSD1* and *JOSD2* genes. This observation implies that the *ATXN3* gene was duplicated after the separation of the Tarsiiformes lineage but before the separation of the Platyrrhini and Catarrhini lineages where an *ATXN3*-like gene is found in every species analyzed. One sequence from *Cavia porcellus* (Rodentia) and one from *Neophocaena asiaeorientalis* (Artiodactyla), unexpectedly cluster with sequences that represent the ancestral *JOSD* gene. In both species, sequences representing the *ATXN3*, *JOSD1* or *JOSD2* genes have been found. Therefore, the misplaced sequences must be a duplicate of either *JOSD1* or *JOSD2*, although the possibility that they are the result of a miss-annotation of the genomes cannot be ruled out. The multiple independent losses of JD containing genes suggests a high degree of functional redundancy in, at least, some lineages.

### 3.2. *Drosophila* interactors bind to ataxin-3 in a similar way to the human interactors

The insights obtained using ataxin-3 transgenic flies (Bilen and Bonini, 2007; Alves et al., 2010; Zhang et al., 2010; Vobfeldt et al., 2012; Xu et al., 2015; Bonini, 2022) are surprising given that in *Drosophila* there is only one gene (*CG3781*, Gene ID 31560, JosL protein), that belongs to the Josephin lineage. *CG3781*, as all other genes from the Josephin lineage, encode a protein that in contrast with ataxin-3 presents a single domain, the catalytic JD (Scheel et al., 2003; Grasty et al., 2019; Supplementary Figure S2). Therefore, the interaction network that is derived using mutant ataxin-3 *Drosophila* could be different from that present in humans. Moreover, even if mutant ataxin-3 *Drosophila* recapitulate the interactions found in humans, it is still possible that the fly proteins interact with human ataxin-3 at different locations than the human ataxin-3 interactors. To address these issues, we compared the ataxin-3 and JosL interactors network, and characterized the interaction regions in human and fly for paralogous interactors.

For ataxin-3, in EvoPPI3 (an aggregator of 12 PPI databases (Vázquez et al., 2018, 2019) updated in June 2022), 423 interactors have been reported (Supplementary Table S4). In *Drosophila* only four proteins are reported as JosL interactors (Supplementary Table S5). Eight (out of 25) of the human homologs of the fly interactors are described as ataxin-3 interactors (Supplementary Table S4). It should be noted that information on modifier genes of mutant ataxin-3 flies is not available in the main PPI databases for *Drosophila*. Therefore, to complete the fly network, we also used the 521, 126, and 18 modifier genes reported in the mutant ataxin-3 screens of Vobfeldt et al. (2012); Supplementary Table S1; Zhang et al. (2010); Supplementary Table S2; and Bilen and Bonini (2007); Supplementary Table S3, respectively. Using DIOPT and Ensembl (section “Materials and Methods”) we obtained the 3,090, 692, and 172 paralogous human genes, respectively.

The human paralogs of 162 fly modifier genes have been shown to encode proteins that are interactors of ataxin-3 (Supplementary Table S6). Therefore, the *Drosophila* model may potentially recapitulate a significant portion (about 38%) of the human ataxin-3 protein interactions. Nevertheless, the number of ataxin-3 interactors present in mutant *Drosophila* could be even higher, since only 49 of the human ataxin-3 interactors do not present a fly paralog, according to DIOPT.

Out of the 162 interactors present in human and flies, 159 are expressed in human basal ganglia, cerebral cortex, midbrain, thalamus, medulla oblongata and/or pons, tissues where *ATXN3* is also expressed, that are relevant for the SCA3 pathology (Seidel et al., 2012; Supplementary Table S6). *Drosophila* interactors are also expressed in brain and/or eye, with the exception of ppk14-PB, uncharacterized proteins Dmel_CG6873 and Dmel_CG5440, and Art6-PA (GeneID 33887, 32861, 33318 and 41699; Supplementary Table S6). Nevertheless, here we considered all these proteins since they present a phenotype when RNAi flies for these genes are crossed with *ATXN3* mutant flies (lethal for genes 33318 and 41699, suppression for 3387, and enhancement for 32861; Vobfeldt et al., 2012). These 162 proteins, according to function, are overrepresented in protein classes such as chaperone (30), protein modifying enzymes (36), protease (24), RNA metabolism (15), ubiquitin-protein ligase (10), translational (10), and cytoskeletal (12) proteins, according to PANTHER Gene Ontology enrichment analysis (Ashburner et al., 2000; Mi et al., 2019; Gene Ontology, 2021).

When applying the *in-silico* methodology (Rocha et al., 2019) for the four JosL interactors, using a cutoff of 50%, 59 amino acid positions (out of 85 amino acid positions that show interaction with at least one of the interactors) are found to be relevant for the interactions (Supplementary Figure S1). When performing this methodology (Rocha et al., 2019) with a subset of 45 fly modifier genes that have human paralogs coding for proteins described as interacting with human ataxin-3 (Table 1; gene ID 43856 and gene ID 34551 were not analyzed due to methodology constrains; see section “Materials and Methods”), and using the same criteria to define interaction regions (amino acid positions that interact with more than 50% of the interactors, larger than three amino acid positions, and that are less than three consecutive amino acids apart from each other) 43 (out of 59 JosL interaction sites) are in common with those used in the interaction with the four interactors reported in EvoPPI3. Indeed, there is a significant overlap of the JosL interacting residues in the two datasets (considering the percentage of interacting residues along the protein (Pearson’s correlation coefficient *R* = 0.9158; *p* < 0 0.00001; y = 1.0222x + 0.3316, being x and y the occupancy frequency of each residue in JosL putative and described interactors, respectively, Supplementary Figure S2). The interaction sites define five regions, and four of them are in the JD (Supplementary Figure S2**)**. Since the JD is highly conserved between the fly JosL and human ataxin-3, this result sugests that the JD region could also be, important for the interaction with human ataxin-3 in mutant *Drosophila*.

**Table 1:** The Ataxin-3 interactors here analyzed, presenting *Drosophila* paralog genes reported as modifiers of polyQ toxicity in mutant flies.

**Ataxin-3 interactor (gene synonyms; GeneID)**	**Paralogous *Drosophila* gene**	**Ataxin-3 interaction**
Transport		
HSPA1A; 3303	39557 (CG6603)	wt and exp forms (Kristensen et al., 2018)
HSPA8; 3312	39557 (CG6603)	wt and exp forms (Kristensen et al., 2018; Weishaupl et al., 2019)
HSPA4L; 22824	39557 (CG6603)	wt and exp forms (Kristensen et al., 2018; Weishaupl et al., 2019)
HSPA4; 3308	39557 (CG6603)	
HSPH1; 10808	39557 (CG6603)	wt and exp forms (Kristensen et al., 2018)
HSP90AA1; 3320	38389 (CG1242)	
DNAJB2; 3300	38643 (CG10578); 31978 (CG2887)	HSJ1 UIM domains binds to ubiquitinated chains on ataxin-3 (Gao et al., 2011)
SLC3A2; 6520	35826 (CG8695)	Mediates aggregation with exp ataxin-3. Overexpression in exp ataxin-3 reverse abnormalities (Paul et al., 2018)
DNAJA1; 3301	41646 (CG8863); 31978 (CG2887); 34984 (CG4599); 38643 (CG10578); 36797 (CG8448)	wt and exp forms (Kristensen et al., 2018; Weishaupl et al., 2019)
SLC25A5; 292	32007 (CG16944)	wt and exp ataxin-3 (Kristensen et al., 2018)
SLC25A6; 293	32007 (CG16944)	wt and exp ataxin-3 (Kristensen et al., 2018)
SLC16A1; 6566	38062 (CG6905)	wt and exp ataxin-3 (Kristensen et al., 2018)
SFXN4; 119559	40552 (CG11739)	wt and exp ataxin-3 (Kristensen et al., 2018)
DNM1L; 10059	31581 (CG3869)	wt and exp ataxin-3 (Kristensen et al., 2018)
ASIC1; 41	33887 (CG9501)	
TOMM20L; 387990	41285 (CG14690)	
Binding		
SERPINH1; 871	49803 (CG10913); 326261 (CG12318/CG33121)	Enriched in exp form comparing with wt form (Kristensen et al., 2018)
DDX39A; 10212	33781 (CG7269)	wt and exp forms (Kristensen et al., 2018)
BAG3; 9531	38151 (CG9153); 37851 (CG4005)	
CANX; 821	44643 (CG11958)	
PARVA; 55742	3772007 (CG33931)	
SRI; 6717	39165 (CG8107)	
Protein cleavage		
CASP1; 834	39173 (CG8091)	
CASP3; 836	43514 (CG7788)	
USP21; 27005	33132 (CG14619)	
CAPN2; 824	39165 (CG8107)	
Transcription activity		
CREBBP; 1387 ^a^	43856 (CG15319)^a^	Exp form binds more effectivetly than the wt form, at the C-terminal region (Li et al., 2002, 2007)
EP300; 2033 ^a^	43856 (CG15319)^a^	Exp form binds more effectively than the wt form, at the C-terminal region (Li et al., 2002)
HDAC3; 8841	38565 (CG7471)	N-terminal ataxin-3 region inhibits histone 4 acetylation (Li et al., 2002)
HDAC6; 10013	38565 (CG7471)	wt form, at the C-terminal region of between residues 319–344 (Bonanomi et al., 2014)
GCAT; 23464	36448 (CG4016)	wt and exp forms (Kristensen et al., 2018)
KAT2B; 8850	43460 (CG14514); 43856 (CG15319)^a^	exp ataxin-3 binds more effectively that the wt form, at the C-terminal region (Li et al., 2002)
Ubiquitination		
SMURF1; 57154	38151 (CG9153); 37851 (CG4005)	
ITCH; 83737	38151 (CG9153); 37851 (CG4005)	Overexpression of ITCH decrease exp ataxin-3 (Chhangani et al., 2014)
UBA1; 7317	35998 (CG1782); 41532 (CG12276); 44496 (CG7528)	Inhibition of UBA1 led to an increase in levels of mutant protein aggregates (Groen and Gillingwater, 2015)
TRAF6; 7189	31746 (CG10961)	
PSMC5; 5705	43635 (CG2241)	wt and exp forms (Wang et al., 2007), at the N-terminal region (Wang et al., 2007)
PSMD4; 5710	40388 (CG7619)	
PSMD7; 5713	34551 (CG4751)^b^	wt and exp forms, at residues 1–150 (Doss-Pepe et al., 2003)
SUMO1; 7341	33981 (CG4494)	wt and exp forms (Zhou et al., 2013)
UBQLN1; 29979	31564 (CG11700)	
UBE2L3; 7332	37035 (CG5788); 33226 (CG3018); 33318 (CG5440)	
UBE2G1; 7326	33226 (CG3018); 33,318 (CG5440); 37035 (CG5788)	wt ataxin-3, but only after using a cross-linking agent DTSSP. Active site C14 is necessary for interaction (Durcan et al., 2012)
UBE2S; 27338	37035 (CG5788); 33226 (CG3018); 33318 (CG5440)	
UBE2N; 7334	37035 (CG5788); 33226 (CG3018); 33318 (CG5440)	
UBB; 7314	31564 (CG11700)	wt and exp forms (Kristensen et al., 2018)
UBC; 7,316	31564 (CG11700)	Exp ataxin-3 C-terminal tail recruits more Ub forms to insoluble aggregates than wt ataxin-3 C-terminal tail (Yang et al., 2014)
RAD23B; 5887	31564 (CG11700)	
RAD23A; 5886	31564 (CG11700)	wt and exp forms, at N-terminal region (Wang et al., 2000). C-terminal region (249–341 aa) of exp form is responsible for recruit of RAD23A to aggregates (Wang et al., 2000)
NEDD8; 4738	31564 (CG11700)	wt form, at the JD (Ferro et al., 2007)
Phosphorylation		
PNKP; 11284	40994 (CG9601)	wt ataxin-3 stimulates PNKP 3′-phosphatase activity, while exp ataxin-3 inhibits PNKP activity (Chatterjee et al., 2015)
CSNK2B; 1460	32132 (CG15224)	phosphorylates wt and exp ataxin-3 at S236, S340 and S352 (Mueller et al., 2009)
NFKBIA; 4792	39375 (CG17153)	
Tubulin related		
TUBB; 203068	37238 (CG9277)	wt form binds to the JD, but not the exp ataxin-3 (Mazzucchelli et al., 2009). TUBB can interact with ataxin-3 also before the polyQ tract (range from residues 244 to 291) and after the polyQ tract (range from residues 319–362) (Bonanomi et al., 2014)
TUBA1A; 7846	37238 (CG9277)	wt ataxin-3 binds to the JD, but not exp ataxin-3 form (Mazzucchelli et al., 2009)
DNM2; 1785	31581 (CG3869)	

a Not analyzed due to I-TASSER size limitation.

b Not analyzed due to HADDOCK error.

**Figure 2:**
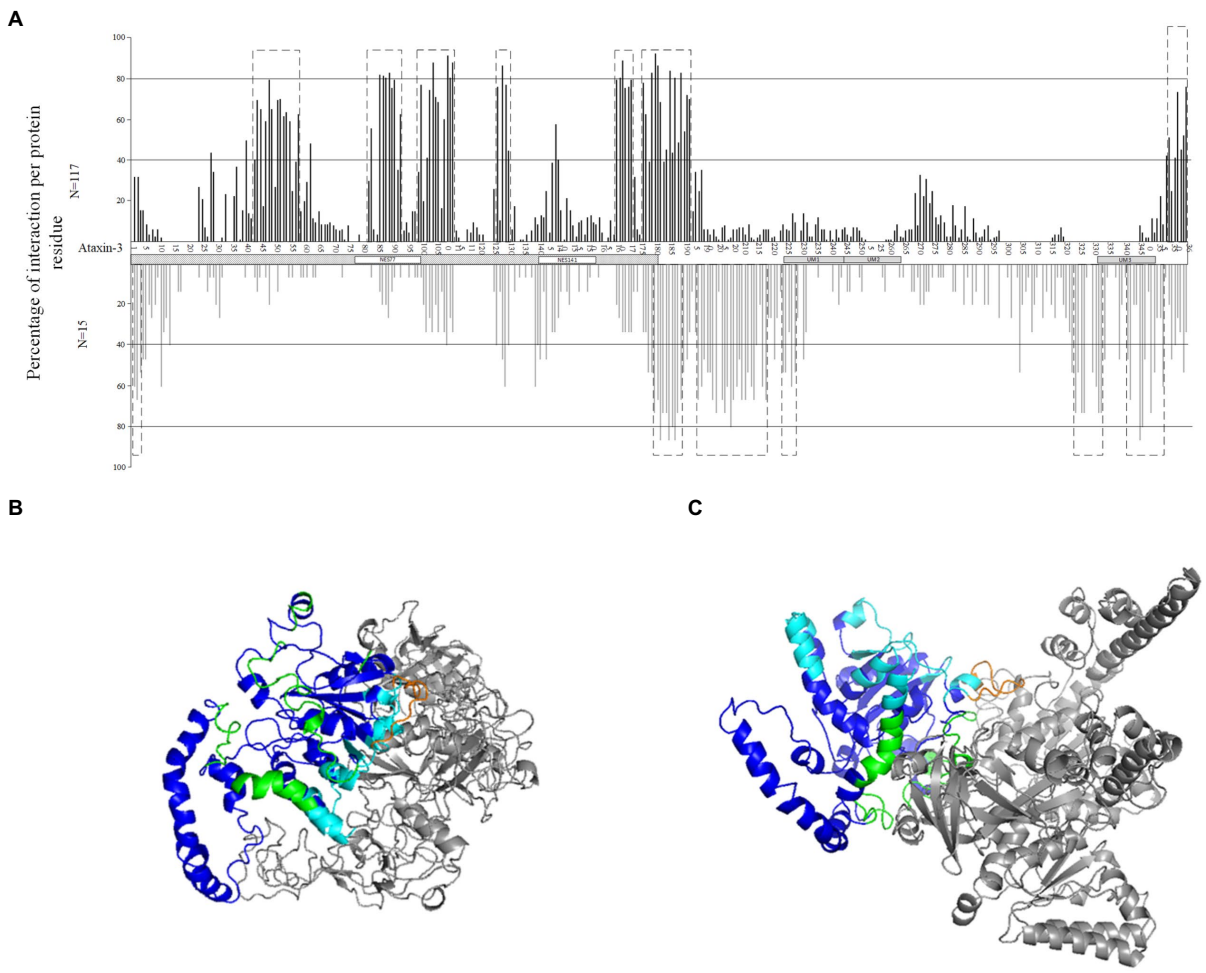
Percentage of interaction per protein residue at the ataxin-3 **(A)**, for the proteins that show most of the interactions at the JD (upper panel, in black) and those that interact mostly at the C- terminal region (lower panel, in gray). Dotted boxes represent regions of interaction where more than 50 percent of the proteins show interaction with ataxin-3. The Josephin domain (JD) is assigned as a dotted box, while the NES77 and NES141 regions are marked with white boxes. UIM regions are also assigned with gray boxes. The dotted boxes ataxin-3 interaction regions are shown on top of the predicted docking structure of ataxin-3 (in dark blue) when interacting with UBC (Gene ID 7316; in gray) that interacts mostly at the JD **(B)**, as well as on top of the predicted docking structure of ataxin-3 (in dark blue) when interacting with VCP (Gene ID 7415; in gray) **(C)** that interacts mostly at the C-terminal region. The interaction regions that show most of the interactions at the JD are marked in light blue and those of the proteins that show most of the interactions at the C- terminal region are in green. In orange is the interaction region in common between the two interaction types.

**Figure 3:**
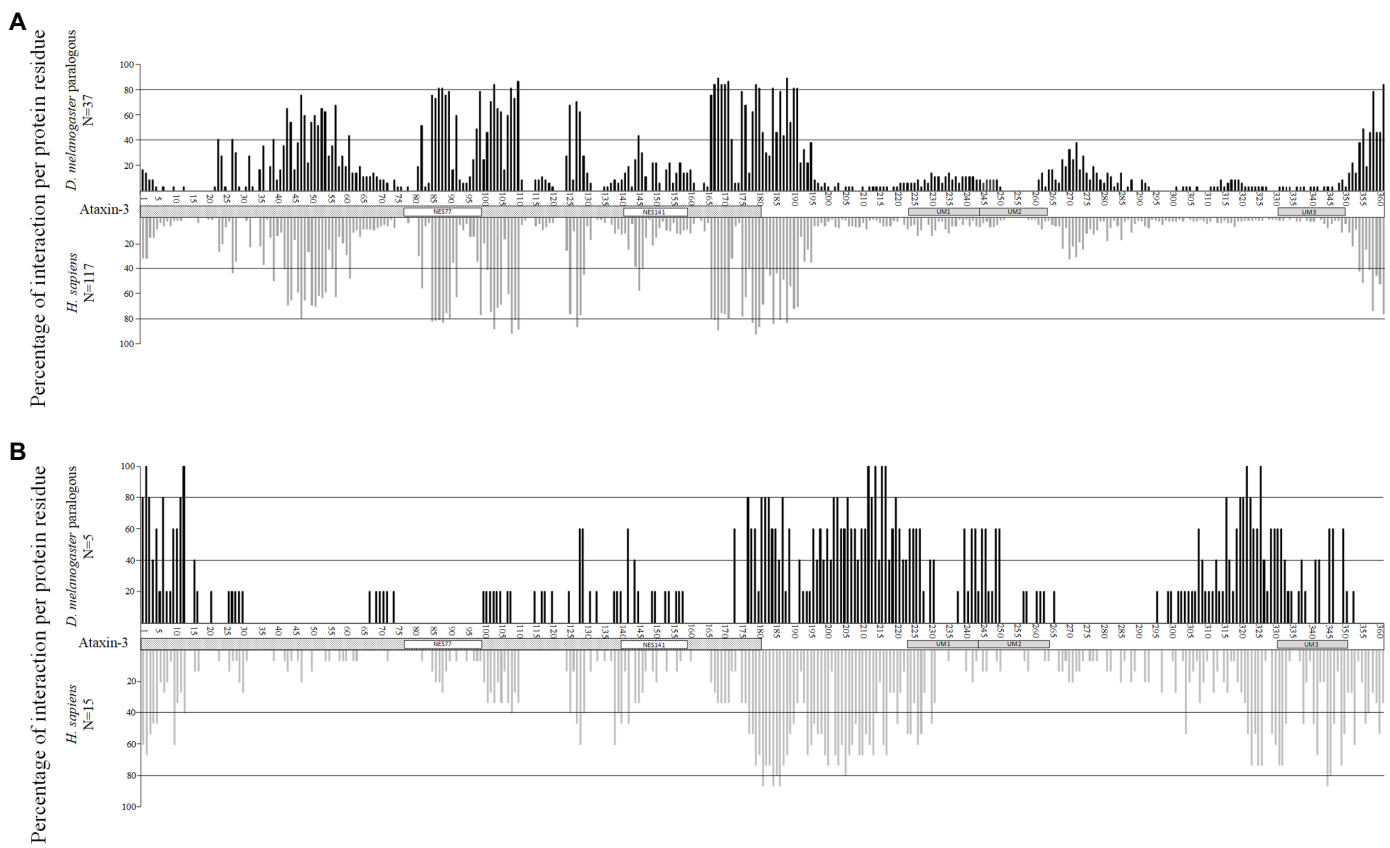
Percentage of interaction per protein residue at the ataxin-3 for fly paralogous (upper panel, in black) and human interactors (lower panel, in gray) that show most of the interactions at the JD **(A)** and those that interact mostly at the C- terminal region **(B)**. The JD is assigned as a dotted box, while the NES77 and NES141 regions are marked with white boxes. UIM regions are also assigned with gray boxes.

When looking at the set of 132 wt ataxin-3 interactors (18 were excluded due to methodology constrains; see section “Materials and Methods”), these proteins show, on average, 65.8% (ranging from 12 to 86%) of the interaction residues at the JD region (Figure 2, upper panel). Since the JD (180 amino acids) is half of the wt-ataxin3 protein (361), we compared the number of interaction residues in these two regions, and there are significantly more interaction residues at the JD than in the remaining region (Sign test; *p* < 0.001). It should be noted that for 117 (out of 132) sequences, there are more interaction residues (70.7%) at JD than at the remaining part of ataxin-3, but for 15 proteins most interactions are found outside the ataxin-3 JD [CDKN1A (Gene ID 1026), DNM2 (Gene ID 1785), EWSR1 (Gene ID 2130), HSP90AA1 (Gene ID 3320), TRAF6 (Gene ID 7189), VCP (Gene ID 7415), PCAF (Gene ID 8850), BAG3 (Gene ID 9531), SLC27A4 (Gene ID 10999), FAM184B (Gene ID 27146), PARVA (Gene ID 55742), Praja1 (Gene ID 64219), SPRTN (Gene ID 83932), HSDL2 (Gene ID 84263), and MCU (Gene ID 90550); Figure 2, lower panel]. This pattern of interaction has been reported for five proteins (Matos et al., 2019) namely, VCP (Gene ID 7415), and PCAF (Gene ID 8850) here analyzed, P300 (Gene ID 2033) and CBP (Gene ID 1387) that are larger than 1,500 amino acids and thus could not be analyzed, and CITED2 (P300/CBP; Gene ID 4435) that is not reported in EvoPPI as an ataxin-3 interactor. Therefore, the correct identification of proteins that bind mostly at the JD region or outside this region validates the *in-silico* predictions. The clusters of interaction sites, identified as above, define molecular recognition patterns that can be used to identify novel ataxin-3 interactors *in-silico*, as well as the interaction type (mostly at the JD or outside this region; Figure 2A). For those interactors that show most of the interaction sites at the JD we identify five molecular recognition regions at the JD, another at the end of the JD and adjacent region, and one at the end of the N-terminal region (Figure 2A upper panel). It should be noted that the number of interactions in the six molecular recognition regions for proteins that show the same subcellular location as ataxin-3 (nucleoplasm, plasma membrane, and nucleoli according to The Human Atlas- Subcellular location data; *N* = 66) and those that show, at present, no evidence for being located in these subcellular locations (*N* = 37) is not different (Mann–Whitney U Test; *p* > 0.05). Thus, the ataxin-3 interactors here studied could interact with ataxin-3 if they have a similar subcellular location. For the set of 15 interactors that show more interaction sites at the C-terminal region of ataxin-3, six molecular recognition regions are identified, namely, one at the JD region, and five at the C-terminal ataxin-3 region (Figure 2A lower panel). The two interaction recognition regions overlap only at the end of the JD and surrounding region (Figures 2A–C for an example of each PPI binding type). Therefore, this region may be crucial for ataxin-3 interaction. The same interaction patterns are observed when considering the binding of fly proteins with human ataxin-3 (Figures 3A,B). For the proteins that interact mostly at the JD we find a significant correlation (Pearson’s correlation coefficient *R* = 0.9763; *p* < 0 0.00001; y = 1.0222x + 0.1672, being x and y the occupancy frequency of each ataxin-3 residue, in *Drosophila* and *Homo*, respectively; Figure 3A) between usage (in percentage) of an amino acid site at the human ataxin-3, when considering human (N = 117) and fly (N = 37) interactors. When considering the interactors that present a larger number of interaction regions outside the JD region, we also find a positive correlation (Pearson’s correlation coefficient R = 0.6804, *p* < 0.00001, y = 0.5904x + 8.3574, being x and y the occupancy frequency of each ataxin-3 residue, in *Drosophila* and *Homo*, respectively; Figure 3B) between usage (in percentage) of an amino acid site at the human ataxin-3 when considering human (N = 15) and fly (N = 5) interactors, despite the small sample size. Since the JosL fly interactors show a similar behavior to the human ataxin-3 interactors, mutant ataxin-3 flies are good models for SCA3.

The inferred ataxin-3 molecular recognition regions seem to be robust against the usage of protein structures that may not be fully accurate. Indeed, the protein fold of 81 proteins that interact mostly at the JD region is similar (TM score higher than 0.5^19^; Supplementary Table S9) when comparing I-TASSER and AlphaFold inferred structures, but for 36 is not (TM score lower than 0.5). Nevertheless, a highly significant correlation (Pearson’s correlation coefficient *R* = 0.968; *p* < 0.00001) is observed between the frequency of usage of an ataxin-3 amino acid site as a binding site in the two datasets. These analyses were not performed for the interactors that interact mostly at the C-terminal ataxin-3 region since the sample size is small (N = 5 and N = 10 for the set of proteins that show the same and different folds when their structure is inferred using I-TASSER or AlphaFold, respectively).

### 3.3. JOS1 and JOS2 can interact with ataxin-3 interactors

The observation that JosL interactors are able to interact with human ataxin-3 in the same way that human ataxin-3 interactors do, raises the question of whether JOS1 and JOS2, that also belong to the Josephin lineage (Figure 1), are able to interact with ataxin-3 interactors. According to Brainspan (Hawrylycz et al., 2012), *ATXN3*, *JOSD1* and *JOSD2* show similar expression levels in the striatum (belongs to basal ganglia) and mediodorsal nucleus of thalamus, two brain tissues that matter to SCA3, along the life time (Supplementary Figure S3), in contrast to *ATX3L*, that is mainly expressed in testis. If Josephin proteins are able to interact with ataxin-3 interactors, this could explain why the inactivation of the *ATXN3* in mouse and *C. elegans* does not lead to gross neurological abnormalities compared with wt animals (Rodrigues et al., 2007; Schmitt et al., 2007).

**Figure 4:**
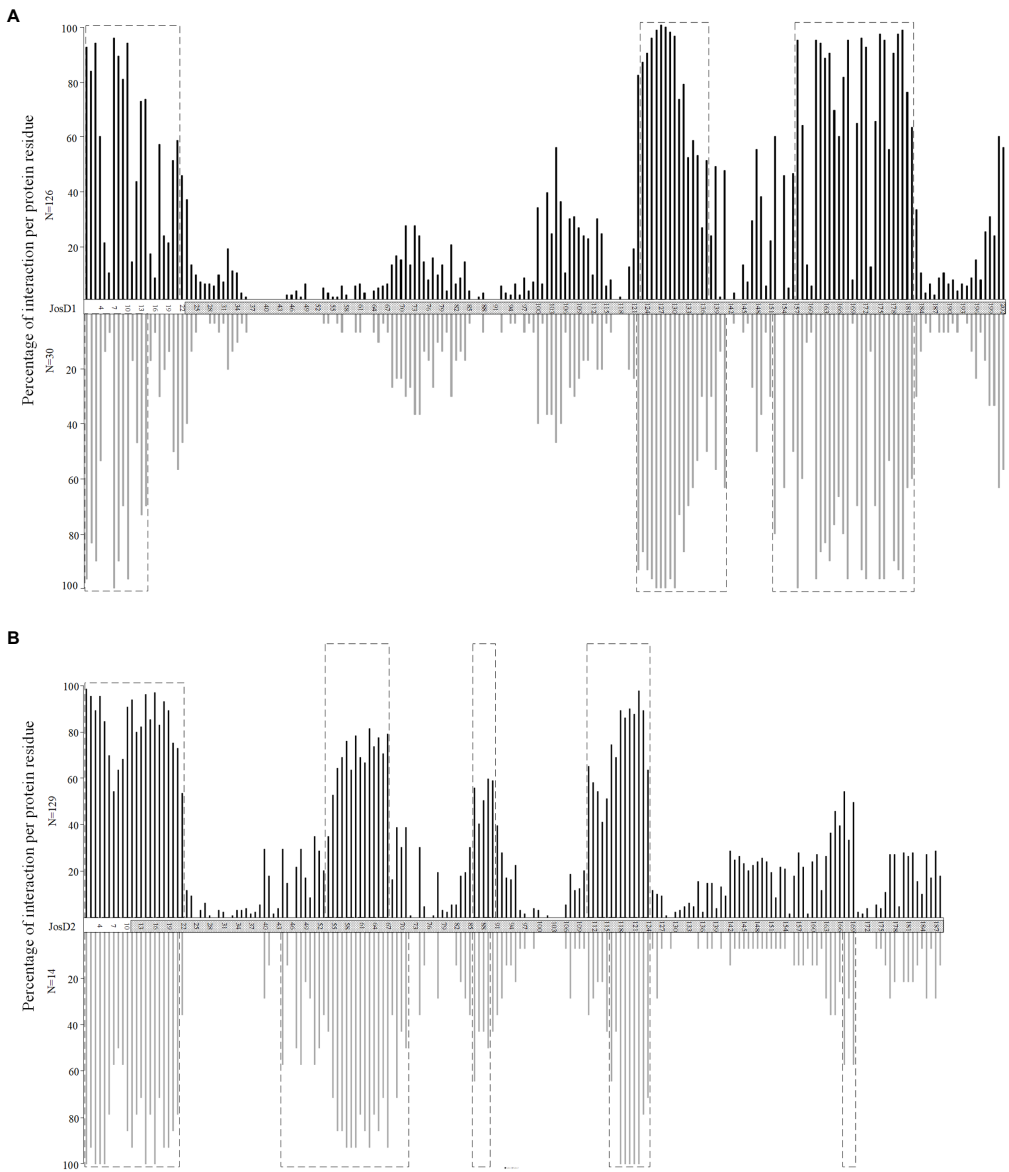
Percentage of interaction per protein residue at the JOS1 **(A)** and JOS2 **(B)** for predicted interactors (those of the ataxin-3; upper panel, in black) and those that are described as the JOS1 and JOS2 interactors (lower panel, in gray), respectively. The interactors in common were excluded from this analysis. Dotted boxes represent regions of interaction where more than 50 percent of the proteins show interaction with JosD1 and JosD2, respectively. The Josephin domain (JD) is assigned as a dotted box.

The JOS1 and JOS2 PPI network obtained from EvoPPI (an aggregator of 12 PPI databases; (Vázquez et al., 2018, 2019), revealed 39 and 17 interactors, respectively (Supplementary Table S7). It is possible that the two networks are incomplete, since no large PPI screen has been performed for these proteins, and few studies (13 for JOS1, and five for JOS2, comparing with 105 for ataxin-3) report PPIs. Comparing the JOS1 and JOS2 networks only two proteins are in common, in agreement with the different functions of these proteins as well as different subcellular locations (JOS1 is preferentially located in the plasma membrane and JOS2 in the cytoplasm; Seki et al., 2013). Nevertheless, when considering the subcellular location, according to The Human Protein Atlas, no data is available for these proteins. For JOS1 interactors, only three (out of 18 for which there is information) are present in the plasma membrane. This suggests that data for plasma membrane location is very incomplete (80% of the data may be missing), and cannot be used as a confirmation set for PPIs. For JOS2, nine interactors (out of 11) are located, as expected, in the cytoplasm. Therefore, although the data for proteins located in the cytoplasm is likely more complete, there is still missing data (18.1%). The comparison of the JOS1 and JOS2 networks with that of ataxin-3, revealed a similar overlap (15 and 12%, respectively; Supplementary Table S7), despite only JOS1 and ataxin-3 sharing plasma membrane location. There are more ataxin-3 interactors located in the cytoplasm (N = 90) than in plasma membrane (N = 13), and thus it seems likely that ataxin-3 is also located in cytoplasm, as stated by Costa Mdo and Paulson (2012). The location overlap of ataxin-3, JOS1, and JOS2, would mean that ataxin-3 interactors may also interact with JOS1 and JOS2. Indeed, using the *in-silico* methodology, we infer that the 132 ataxin-3 interactors (excluding the ones common to JOS1 or JOS2 network) are able to interact with both JOS1 (*N* = 126) and JOS2 (*N* = 129) and in a similar way to that of JOS1 and JOS2 interactors (for JOS1 considering the percentage of interacting residues along protein, Pearson’s correlation coefficient *R* = 0.9862; *p* < 0 0.00001; y = 1.0043x + 0.8839, being x and y the occupancy frequency of each residue in JOS1 putative and described interactors, respectively, Figure 4A; for JOS2, Pearson’s correlation coefficient *R* = 0.9462; *p* < 0 0.00001; y = 1.047x – 1.9793, being x and y the occupancy frequency of each residue, in putative and described JOS2 interactors, respectively, Figure 4B). Moreover, the number of interactions at the four JOS1 interacting regions is not significantly different from the ataxin-3 interactors with location in the plasma membrane and those with different subcellular locations (Mann–Whitney test; *p* > 0.05). A similar observation is obtained for JOS2 when considering ataxin-3 interactors located in the cytoplasm versus those that are located in other cellular regions (Mann–Whitney test; *p* > 0.05). When location of the JOS1 and JOS2 interacting regions is compared, two are in common but none overlaps with those of wt ataxin-3 (Supplementary Figure S4). Binding at these regions could, however, contribute for the partial phenotype rescue observed in mutant mouse and *C. elegans* without ataxin-3 (Rodrigues et al., 2007; Schmitt et al., 2007).

### 3.4. Interaction regions at ataxin-3 are affected by the presence of an expanded polyglutamine

SCA3 pathology is associated with an expanded polyQ tract in ataxin-3 protein (Matos et al., 2019; McLoughlin et al., 2020). The expanded polyQ tract leads to structural changes of the translated protein, that alters native PPIs, and thus, the normal protein activity (Lim et al., 2006; Hosp et al., 2015; Rocha et al., 2019). It should be noted that the polyQ is located between a disordered region and a coiled-coil region used for PPI (Schaefer et al., 2012; Mier and Andrade-Navarro, 2021). It has been hypothesized that when expanded, the polyQ region would adopt a helical conformation, extending the preceding helix, and thus making the PPI interactions stronger (Schaefer et al., 2012; Petrakis et al., 2013; Mier and Andrade-Navarro, 2021). For instance, VCP delays exp ataxin-3 for proteasome degradation, because exp ataxin-3 is not dissociated from E4B protein (that endorses ataxin-3 for degradation; Matsumoto et al., 2004). This could reveal differences in interactions conferred by the polyQ expansion, which in turn may indicate aberrant interactions implicated in disease. Identifying the proteins that show a different behavior with the exp polyQ ataxin-3, is thus, fundamental for understanding SCA3.

**Figure 5:**
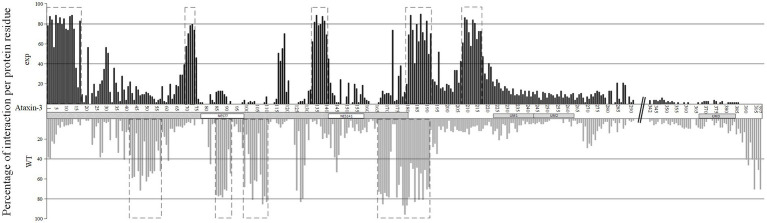
Percentage of interaction per protein residue at the exp (upper panel, in black) and wt (lower panel, in gray) ataxin-3 forms. Dotted boxes represent regions of interaction where more than 50 percent of the proteins show interaction with the two ataxin-3 forms. The Josephin domain (JD) is assigned as a dotted box, while the NES77 and NES141 regions are marked with white boxes. UIM regions are also assigned with gray boxes. The polyQ region is assigned with //.

**Figure 6:**
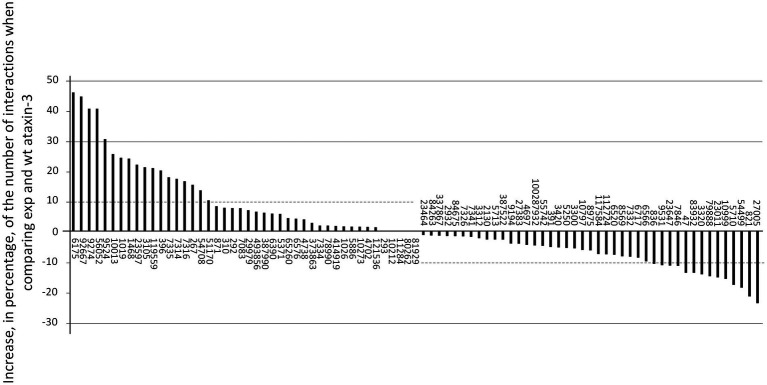
Increase, in percentage, of the number of interactions when comparing exp and wt ataxin-3 forms for 87 interactors, assigned as GeneIDs.

To address differences in binding strength between wt and exp ataxin-3, *in-silico* analyses were performed using the 132 ataxin-3 interactors and exp ataxin-3. Nevertheless, 45 proteins show six or more interactions in the polyQ region (these could be anomalous predictions (Rocha et al., 2019; Supplementary Table S8), and thus only 87 interactors could be analyzed (that include eight of the 15 proteins that show more interactions at the C-terminal region; Figure 5). When considering the total number of interactions, there is no tendency for an increased number of interactions in the exp ataxin-3 when compared with the wt ataxin-3 form (Sign test *p* > 0.05; N = 87; Positive differences (exp – wt) = 39; Negative differences = 43; Ties = 5). On average, there are 67.3 and 68.1 interfacing residues for ataxin-3 wt and exp., respectively. This suggests that the exp polyQ does not affect equally the binding strength for all ataxin-3 interactors, as expected. Indeed, some interactors are described as binding in a similar way to both ataxin-3 forms as for instance SUMO1 (GeneID 7341; Table 1), as here also observed. As expected under the above hypothesis, 20.7% of the interactors show an increase larger than 10% in the number of interaction residues in exp ataxin-3 relative to the wt form, and these proteins may be relevant for SCA3 (Figure 6). These 18 proteins are, according to PANTHER cellular component classification system, significantly enriched in extrinsic components of mitochondrial outer membrane (UBB, 7314; and UBC, 7316) and endoplasmatic reticulum membrane (UBC, 7316; GP78, 267; ALG1, 56052; UBB, 7314; TECR, 9524; MARCH5, 54708; and HLA-A, 3105). This is in agreement with the literature, since the role of mitochondrial dysfunction is well established in polyQ diseases (Laço et al., 2012; Guedes-Dias et al., 2015). Moreover, ataxin-3 is involved in the degradation of misfolded proteins by the endoplasmic reticulum-associated protein degradation system (Wang et al., 2006; Zhong and Pittman, 2006). It should be noted, however, that none of the five mitochondrial interactors suggested as significantly enriched in the binding with exp ataxin-3 (Kristensen et al., 2018) are here identified as showing more interactions with exp ataxin-3. Nevertheless these proteins have been identified based on immunoprecipitation experiments coupled with LC–MS/MS analyses using HEK293 cells expressing the two ataxin-3 forms, and have not been confirmed using other approaches (Kristensen et al., 2018), and could be artifacts of the methodology used.

Moreover, 16.1% of the interactors show a decrease larger than 10% in the number of interaction residues in exp ataxin-3 relative to the wt form (Figure 6), and these proteins may also be relevant for SCA3. These 14 proteins are, according to PANTHER Go-slim cellular component classification system, significantly enriched in extrinsic components of cytoplasm (HSP90AA1, 3320; RAB21, 23011; PSMD4, 5710; SLC27A4, 10999; ARFIP2, 23647; ACLY, 47; CASP-3, 836; USP21, 27005; TUBA1A, 7846; TMCO1, 54499; BAG-3, 9531; CANX, 821). This could suggest that polyQ-induced pathogenesis is primarily activated in the cytoplasm.

When considering the JD only, there is a tendency for wt ataxin-3 to have more interactions than exp ataxin-3 (Sign test *p* < 0 0.00001; *N* = 87; Positive differences (exp – wt) = 19; Negative differences = 65; Ties = 3; Figure 5). On average, there are 45.2 and 39.4 interfacing residues for ataxin-3 wt and exp., respectively at the JD. Moreover, as expected since the total number of interactions is similar for the two ataxin-3 forms, when considering the C-terminal region only, there is a tendency for wt ataxin-3 to have less interactions than exp ataxin-3 (Sign test *p* < 0 0.00001; *N* = 87; Positive differences = 67 (exp – wt); Negative differences = 19; Ties = 1; Figure 5). On average, there are 22.1 and 28.7 interfacing residues for ataxin-3 wt and exp, respectively in the C-terminal region. This pattern is in agreement with previous reports (see for instance UBC (7316), RAD23A (5886), NEDD8 (4738), TUBA1A (7846) in Table 1, and here studied). Thus, it is not surprising that, when considering clusters of interaction sites (defined as above) no overlap is observed between wt and exp ataxin-3 interaction signature, except in the region after the JD (Figure 6).

By comparing the interaction sites at exp ataxin-3 between human (*N* = 87) and fly homologous interactors (*N* = 25; 18 interactors show six or more interactions at the polyQ region, and thus were removed), we also find a similar correlation (Pearson’s correlation coefficient *R* = 0.9737; *p* < 0.00001; y = 0.8933x + 3.2223, being x and y the occupancy frequency of each ataxin-3 residue, in *Drosophila* and *Homo*, respectively). Moreover, when considering clusters of interaction sites of fly interactors with exp ataxin-3, we find the same five regions as show in Figure 6 for human interactors with exp ataxin-3, plus one (between sites 117–119; Supplementary Figure S5). This is why *Drosophila* can be used as a SCA3 model.

## 4. Discussion

The multiple losses of JD containing genes observed across the animal tree suggest that there is a high degree of redundancy of the function of these genes. Nevertheless, in all animal groups for which a large number of genomes is available, with the clear exception of Lepidoptera and Diptera, there is always one representative of each lineage, suggesting that the redundancy is only partial. *Drosophila* mutants expressing human *ATXN3* have been used as a model for SCA3 research disorder (Bilen and Bonini, 2007; Alves et al., 2010; Zhang et al., 2010; Vobfeldt et al., 2012; Xu et al., 2015; Bonini, 2022). Nevertheless, as here shown, there is no *ATXN3* gene lineage in *Drosophila*. Therefore, it was of interest to understand whether this is a good model for SCA3, namely if the PPI interactions observed in human and in the mutant *Drosophila* model are the same, and whether *Drosophila* proteins bind the human ataxin-3 protein the same way as their human homologs do. Here we show an overlap of 38% of the PPI networks observed in humans and the ones that could be inferred in mutant *Drosophila* using large scale modifier screens [521 (Vobfeldt et al., 2012); 126 (Zhang et al., 2010); 18 (Bilen and Bonini, 2007) genes]. This is a high number, since not all modifiers are expected to be ataxin-3 interactors. Nevertheless, 62% of all human interactors have not been identified using this approach. Moreover, the human ataxin-3 network may also be incomplete, and thus, the overlap of the two networks could be much higher than here estimated. Furthermore, here we inferred that the proteins of paralog fly genes show similar molecular recognition regions to those described in human. Therefore, information on distantly related model organisms can be used to complete the ataxin-3 network. This characterization is essential to interpret the large perturbations identified in transcriptomic and proteomic analyses using patient tissues and animal models (Toonen et al., 2018; Wiatr et al., 2019; Sowa et al., 2021; Haas et al., 2022).

According to binding preferences, ataxin-3 interactors can be divided in two groups: those (more than 88%) that interact mostly at the JD and a small set that interacts most at the C-terminal ataxin-3 region. The interaction regions identified as essential for ataxin-3 binding have been identified using an unbiased sample of interactors. The use of a larger dataset will allow to address associations of these regions with functional classes of ataxin-3 interactors. The observation that *Drosophila* homologs show similar interaction regions with ataxin-3 can explain why mutant wt *ATXN3* flies do not show signs of neurodegeneration, in contrast with the exp *ATXN3* mutants (Bilen and Bonini, 2007; Alves et al., 2010; Zhang et al., 2010; Vobfeldt et al., 2012; Xu et al., 2015; Bonini, 2022).

The inference that ataxin-3 JD is essential for binding with a large number of the wt ataxin-3 interactors, suggests that these proteins could also interact with JOS1 and JOS2. Indeed, it is here predicted that the 126 and 129 ataxin-3 interactors can bind JOS1 and JOS2, respectively, if these interactors show similar subcellular location as JOS1 and JOS2. Nevertheless, the molecular recognition regions of ataxin-3 binding and those predicted for the Josephins are different, suggesting that the binding strength could be different. This result may explain why mouse and nematode knockout *ATXN3* models reveal no neurodegenerative phenotype (Costa Mdo and Paulson, 2012).

The ataxin-3 structural changes associated with the expanded polyQ alters native PPIs, that causes SCA3 pathology (Lim et al., 2006; Hosp et al., 2015; Rocha et al., 2019). Considering the network of 87 ataxin-3 interactors here analyzed, we infer, that not all proteins are equally affected by the expanded polyQ. Considering the difference on the number of interaction sites between exp and wt ataxin-3, normalized by the number of wt interactions, as a measure of interaction strength, 62% of the interactors likely have a similar interaction strength (less than 10% difference) with the two ataxin-3 forms. Nevertheless, there are 18 interactors inferred to have an increase in the interaction strength with exp ataxin-3, that are enriched in extrinsic components of mitochondrial outer membrane and endoplasmatic reticulum membrane, as observed before (Wang et al., 2006; Zhong and Pittman, 2006; Laço et al., 2012; Guedes-Dias et al., 2015). MITOL, a mitochondrial ubiquitin ligase here identified as interacting significantly more with exp ataxin-3, localized in the mitochondrial outer membrane is involved in the degradation of pathogenic ataxin-3 in mitochondria (Sugiura et al., 2011). Ataxin-3 is also involved in the degradation of misfolded proteins by the endoplasmic reticulum-associated protein degradation system (Wang et al., 2006; Zhong and Pittman, 2006), and thus it is not surprising that proteins of this system could interact more with exp ataxin-3.

Moreover, for 15 interactors we predict a decrease larger than 10% in the number of interaction residues in exp ataxin-3 relative to the wt form. This group of proteins are significantly enriched in extrinsic component of cytoplasm, and this could suggest that polyQ-induced pathogenesis is primarily activated in the cytoplasm. These ataxin-3 interactions should by studied in detail using biochemical experiments.

When comparing the number of interactions at the JD and at the C-terminal region between the exp and wt ataxin-3 forms more than 83% of the proteins show an increase in the number of interactions in the C-terminal region, in agreement with the literature (see Table 1 for references). It would be interesting to address if this pattern is also observed in other ataxin proteins, that cause polyQ neurodegenerative diseases. Therefore, the *in-silico* methodology here used is an important tool to predict protein interaction signatures, even when an expanded polyQ tract is present. Further improvements can be made, by using AlphaFold (Tunyasuvunakool et al., 2021) prediction tool to obtain the interactors protein structure. This way limitations concerning protein size as those of I-TASSER (Roy et al., 2010), will be overcome.

## Data availability statement

The datasets presented in this study can be found in online repositories. The names of the repository/repositories and accession number(s) can be found in the article/Supplementary material.

## Author contributions

JV and CV designed the experiments. RS and AS performed the protein structures analyses. All authors analyzed the data, wrote, read and approved the final manuscript.

## Funding

This work was financed by the National Funds through FCT—Fundação para a Ciência e a Tecnologia, I.P., under the project UIDB/04293/2020.

## Conflict of interest

The authors declare that the research was conducted in the absence of any commercial or financial relationships that could be construed as a potential conflict of interest.

## Publisher’s note

All claims expressed in this article are solely those of the authors and do not necessarily represent those of their affiliated organizations, or those of the publisher, the editors and the reviewers. Any product that may be evaluated in this article, or claim that may be made by its manufacturer, is not guaranteed or endorsed by the publisher.

## Footnotes

^1^ https://pegi3s.github.io/dockerfiles/

^2^ https://www.uniprot.org/

^3^ https://github.com/pegi3s/dockerfiles/tree/master/emboss/6.6.0

^4^ https://github.com/pegi3s/dockerfiles/tree/master/clustalomega/1.2.4

^5^ https://github.com/pegi3s/dockerfiles/tree/master/translatorx

^6^ https://github.com/pegi3s/dockerfiles/tree/master/fasttree/2.1.10

^7^ http://evoppi.i3s.up.pt/dashboard

^8^ https://github.com/sing-group/evoppi-backend

^9^ https://www.flyrnai.org/diopt

^10^ http://bioinformatics.psb.ugent.be/webtools/Venn/

^11^ https://zhanglab.ccmb.med.umich.edu/I-TASSER/

^12^ http://milou.science.uu.nl/services/CPORT

^13^ http://milou.science.uu.nl/services/HADDOCK2.2/

^14^ https://www.ebi.ac.uk/pdbe/pisa/

^15^ https://github.com/pegi3s/dockerfiles/blob/master/utilities/scripts/pisa_xml:extract

^16^ https://github.com/pegi3s/dockerfiles/tree/master/pymol

^17^ https://www.proteinatlas.org/humanproteome/brain/human+brain

^18^ http://flyatlas.gla.ac.uk/FlyAtlas2/index.html

^19^ https://zhanggroup.org/TM-score/
