# Supplementary material for: The Josephin domain (JD) containing proteins are predicted to bind to the same interactors: Implications for spinocerebellar ataxia type 3 (SCA3) studies using Drosophila melanogaster mutants
Source: Front Mol Neurosci. 2023 Mar 15;16:1140719. doi: 10.3389/fnmol.2023.1140719 (PMC10050893; doi:10.3389/fnmol.2023.1140719)
Supplement: Supplementary file 7 [file Table_7.DOCX]

**Supplementary Table 7**. The Jos1 and Jos2 interactor in EvoPPI. Stars indicate common ataxin-3 interactors. Proteins marked with a plus signal have not been studied because of the *in-silico* limitations.

|  | Gene ID | Uniport ID | Protein names |
| --- | --- | --- | --- |
| Jos1 and Jos2 |  |  |  |
|  | 7316* | P0CG48 | Polyubiquitin-C (UBC) |
|  | 10273* | Q9UNE7 | E3 ubiquitin-protein ligase CHIP (CHIP) |
| Jos1 | 373 | P36406 | E3 ubiquitin-protein ligase TRIM23 (TRIM23) |
|  | 801 | P0DP23 | Calmodulin-1 (CALM1) |
|  | 805 | P0DP24 | Calmodulin-2 (CALM2) |
|  | 808 | P0DP25 | Calmodulin-3 (CALM3) |
|  | 1678 | O60220 | Mitochondrial import inner membrane translocase subunit Tim8 A (TIMM8A) |
|  | 1994 | Q15717 | ELAV-like protein 1 (ELAVL1) |
|  | 2199+ | P98095 | Fibulin-2 (FBLN2) |
|  | 3846 | P26371 | Keratin-associated protein 5-9 (KRTAP5-9) |
|  | 3881 | Q15323 | Keratin, type I cuticular Ha1 (KRT31) |
|  | 3892 | O43790 | Keratin, type II cuticular Hb6 (KRT86) |
|  | 4188 | Q99750 | MyoD family inhibitor (MDFI) |
|  | 5987 | P14373 | Zinc finger protein RFP (TRIM27) |
|  | 7051 | P22735 | Protein-glutamine gamma-glutamyltransferase K (TGM1) |
|  | 7185 | Q13077 | TNF receptor-associated factor 1 (TRAF1) |
|  | 8665 | O00303 | Eukaryotic translation initiation factor 3 subunit F (EIF3F) |
|  | 9958 | Q9Y4E8 | Ubiquitin carboxyl-terminal hydrolase 15 (USP15) |
|  | 11043 | Q9UJV3 | Probable E3 ubiquitin-protein ligase MID2 (MID2) |
|  | 23281+ | Q5JR59 | Microtubule-associated tumor suppressor candidate 2 (MTUS2) |
|  | 27005* | Q9UK80 | Ubiquitin carboxyl-terminal hydrolase 21 (USP21) |
|  | 29775 | Q9BWT7 | Caspase recruitment domain-containing protein 10 (CARD10) |
|  | 57159* | Q9BYV2 | Tripartite motif containing protein 54 (MURF3) |
|  | 57646 | Q96RU2 | biquitin carboxyl-terminal hydrolase 28 (USP28) |
|  | 64219* | Q8NG27 | E3 ubiquitin-protein ligase Praja-1 (PRAJA-1) |
|  | 81851 | Q07627 | Keratin-associated protein 1-1 (KRTAP1-1) |
|  | 83899 | Q9BYQ4 | Keratin-associated protein 9-2 (KRTAP9-2) |
|  | 83900 | Q9BYQ3 | Keratin-associated protein 9-3 (KRTAP9-3) |
|  | 84676* | Q969Q1 | E3 ubiquitin-protein ligase TRIM63 (MURF1) |
|  | 84708 | Q8TBB1 | E3 ubiquitin-protein ligase LNX (LNX1) |
|  | 125115 | Q6A162 | Keratin, type I cytoskeletal 40 (KRT40) |
|  | 342574 | Q7Z3Y8 | Keratin, type I cytoskeletal 27 (KRT27) |
|  | 375791 | A8MQ03 | Cysteine-rich tail protein 1 (CYSRT1) |
|  | 386672 | P60372 | Keratin-associated protein 10-4 (KRTAP10-4) |
|  | 386681 | P60410 | Keratin-associated protein 10-8 (KRTAP10-8) |
|  | 386682 | P60369 | Keratin-associated protein 10-3 (KRTAP10-3) |
|  | 386683 | P60328 | Keratin-associated protein 12-3 (KRTAP12-3) |
|  | 388677 | Q7Z3S9 | Notch homolog 2 N-terminal-like protein A (NOTCH2NLA) |
| Jos2 |  |  |  |
|  | 3043 | P68871 | Hemoglobin subunit beta (HBB) |
|  | 5320 | P14555 | Phospholipase A2, membrane associated (PLA2G2A) |
|  | 6399 | P0DI81 | Trafficking protein particle complex subunit 2 (TRAPPC2) |
|  | 6595+ | P51531 | Probable global transcription activator SNF2L2 (SMARCA2) |
|  | 6707 | Q9UBC9 | Small proline-rich protein 3 (SPRR3) |
|  | 10768 | O43865 | S-adenosylhomocysteine hydrolase-like protein 1 (AHCYL1) |
|  | 10897 | O95070 | Protein YIF1A (YIF1A) |
|  | 23382 | Q96HN2 | Adenosylhomocysteinase 3 (AHCYL2) |
|  | 23412 | Q9UBI1 | COMM domain-containing protein 3 (COMMD3) |
|  | 116173 | Q96DZ9 | CKLF-like MARVEL transmembrane domain-containing protein 5 (CMTM5) |
|  | 199675 | Q8IX19 | Mast cell-expressed membrane protein 1 (MCEMP1) |
|  | 219743 | Q2T9J0 | Peroxisomal leader peptide-processing protease (TYSND1) |
|  | 220074 | Q8WZ04 | Transmembrane O-methyltransferase (TOMT) |
|  | 285193 | Q4G0W2 | Dual specificity phosphatase 28 (DUSP28) |
|  | 120356739 | LRRC51 | Leucine rich repeat containing 51 |
