# Supplementary material for: The Josephin domain (JD) containing proteins are predicted to bind to the same interactors: Implications for spinocerebellar ataxia type 3 (SCA3) studies using Drosophila melanogaster mutants
Source: Front Mol Neurosci. 2023 Mar 15;16:1140719. doi: 10.3389/fnmol.2023.1140719 (PMC10050893; doi:10.3389/fnmol.2023.1140719)
Supplement: Supplementary file 8 [file Table_8.DOCX]

**Supplementary Table 8.** Proteins that present six or more interactions at polyQ tract in the *in-silico* analyses.

| Subsets analysed | Protein name (Gene ID) |
| --- | --- |
| wt ataxin-3 | RAD23B (5887); DNAJA1 (3301); HSPA4L (22824); APP (351); BCL2L1 (598); PSRC1 (84722); ARHGAP19 (84986); RPS6KA1 (6195); GNPAT (8443) |
| exp ataxin-3 | HSPA4L (22824); HSPA4 (3308); HDAC3 (8841); PCAF (8850); CK2 (1460); NFKBIA (4792); CAPN2 (824); CASP-1 (834); ASIC1 (41); DNM2 (1785); TUBB (203068); TRAF6 (7189); SMURF1 (57154); PSMC5 (5705); DNM1L (10059); HSPH1 (10808); HSPA1A (3303); UBA1 (7317); PARKIN (5071); P53 (7157); VCP (7415); p62 (8878); HRD1 (84447); CREB1 (1385); FOXO4 (4303); MURF3 (57159); SDC2 (6383); KCTD10 (83892); MURF1 (84676); PICK1 (9463); TELO2 (9894); PSRC1 (84722); ARHGAP19 (84986); KU70 (2547); RPL6 (6128); HAP1 (9001); MGRN1 (23295); CNOT6 (57472); PRAJA-1 (64219); TEX11 (56159); MALT1 (10892); IBA57 (200205); FAM184B (27146); SDHA (6389); SLC25A22 (79751); RMDN1 (51115); PRPS1 (5631); MCU (90550) |
