# Supplementary material for: The Josephin domain (JD) containing proteins are predicted to bind to the same interactors: Implications for spinocerebellar ataxia type 3 (SCA3) studies using Drosophila melanogaster mutants
Source: Front Mol Neurosci. 2023 Mar 15;16:1140719. doi: 10.3389/fnmol.2023.1140719 (PMC10050893; doi:10.3389/fnmol.2023.1140719)
Supplement: Supplementary file 9 [file Table_9.docx]

**Supplementary Table 9**. Similarity of the ITASSER and AlphaFold protein structures according to the TM-score (Zhang *et al*. 2005).

| Gene ID | Protein Name | Uniprot ID | TM-score |
| --- | --- | --- | --- |
| 5631 | PRPS1 | P78348 | 0,98100 |
| 203068 | TUBB | P53396 | 0,97696 |
| 293 | SLC25A6 | P05141 | 0,97130 |
| 292 | SLC25A5 | P12236 | 0,96848 |
| 7332 | UBE2L3 | P20073 | 0,96814 |
| 7317 | UBA1 | P84095 | 0,96045 |
| 7326 | UBE2G1 | P52565 | 0,95683 |
| 396 | ARHGDIA | P27824 | 0,95322 |
| 7334 | UBE2N | P17655 | 0,95083 |
| 78990 | OTUB2 | P29466 | 0,94920 |
| 23464 | GCAT | P42574 | 0,94250 |
| 7846 | TUBA1A | P50454 | 0,94231 |
| 1019 | CDK4 | P11802 | 0,93528 |
| 6389 | SDHA | P67870 | 0,92353 |
| 391 | RHOG | Q9UBX3 | 0,92254 |
| 4702 | NDUFA8 | P50570 | 0,92216 |
| 7335 | UBE2V1 | P12956 | 0,90021 |
| 47 | ACLY | P49841 | 0,89977 |
| 1460 | CSNK2B | P0DMV8 | 0,89520 |
| 4738 | NEDD8 | P34932 | 0,89262 |
| 824 | CAPN2 | P11142 | 0,88781 |
| 3303 | HSPA1A | P07900 | 0,88189 |
| 2932 | GSK3B | O43837 | 0,88151 |
| 871 | SERPINH1 | O00483 | 0,88116 |
| 3312 | HSPA8 | P51970 | 0,88080 |
| 3320 | HSP90AA1 | Q15843 | 0,86861 |
| 8841 | HDAC3 | P25963 | 0,86626 |
| 3420 | IDH3B | O60260 | 0,86368 |
| 6390 | SDHB | Q00325 | 0,86052 |
| 10797 | MTHFD2 | P60891 | 0,86009 |
| 200205 | IBA57 | P62195 | 0,85819 |
| 5071 | PRKN | P55036 | 0,84517 |
| 112724 | RDH13 | P51665 | 0,83512 |
| 8975 | USP13 | Q02878 | 0,83424 |
| 2547 | XRCC6 | P05388 | 0,83363 |
| 81929 | SEH1L | P31040 | 0,82084 |
| 6717 | SRI | P21912 | 0,81826 |
| 65260 | COA7 | P08195 | 0,79252 |
| 79751 | SLC25A22 | P53007 | 0,77992 |
| 41 | ASIC1 | P30626 | 0,76918 |
| 836 | CASP3 | P04183 | 0,76672 |
| 11284 | PNKP | P04637 | 0,76097 |
| 10999 | SLC27A4 | P0CG47 | 0,75886 |
| 23011 | RAB21 | P22314 | 0,75663 |
| 6576 | SLC25A1 | P62253 | 0,75561 |
| 7415 | VCP | P68036 | 0,75186 |
| 7083 | TK1 | P61088 | 0,75176 |
| 56052 | ALG1 | Q13404 | 0,74285 |
| 5705 | PSMC5 | P63165 | 0,73772 |
| 5713 | PSMD7 | P55072 | 0,73690 |
| 4697 | NDUFA4 | Q71U36 | 0,73199 |
| 3308 | HSPA4 | Q9BUB5 | 0,72099 |
| 10808 | HSPH1 | O15379 | 0,71935 |
| 51170 | HSD17B11 | Q92995 | 0,71730 |
| 6128 | RPL6 | Q9NZ01 | 0,70708 |
| 51115 | RMDN1 | Q9UBN7 | 0,69203 |
| 7341 | SUMO1 | O00429 | 0,68809 |
| 84263 | HSDL2 | O00148 | 0,68428 |
| 27338 | UBE2S | Q9UNE7 | 0,67888 |
| 23597 | ACOT9 | P13995 | 0,67750 |
| 10059 | DNM1L | Q92598 | 0,67036 |
| 9524 | TECR | Q6P1M0 | 0,65749 |
| 821 | CANX | Q96T60 | 0,65650 |
| 834 | CASP1 | Q9UL25 | 0,65448 |
| 6175 | RPLP0 | O75600 | 0,64532 |
| 310 | ANXA7 | Q9Y305 | 0,63627 |
| 10013 | HDAC6 | P53365 | 0,63131 |
| 4792 | NFKBIA | Q9UK80 | 0,62985 |
| 1468 | SLC25A10 | Q16763 | 0,62407 |
| 1785 | DNM2 | Q96DB5 | 0,61062 |
| 10212 | DDX39A | Q8NBQ5 | 0,60286 |
| 23647 | ARFIP2 | Q9BT22 | 0,59924 |
| 7314 | UBB | Q9BYV2 | 0,59846 |
| 57472 | CNOT6 | Q9ULM6 | 0,59186 |
| 92667 | MGME1 | Q96BR5 | 0,58256 |
| 6520 | SLC3A2 | Q96DC9 | 0,55552 |
| 387522 | PEDS1 | Q9H936 | 0,55471 |
| 10273 | STUB1 | Q96EE3 | 0,55186 |
| 84676 | TRIM63 | Q6YN16 | 0,54679 |
| 27005 | USP21 | Q969Q1 | 0,54444 |
| 8569 | MKNK1 | Q9BQP7 | 0,53991 |
| 5250 | SLC25A3 | Q8NBN7 | 0,53479 |
| 7157 | TP53 | Q5T440 | 0,53100 |
| 5710 | PSMD4 | P07437 | 0,52707 |
| 57159 | TRIM54 | A5PLL7 | 0,52583 |
| 414919 | C8orf82 | Q6P1X6 | 0,50698 |
| 493856 | CISD2 | Q8N5K1 | 0,49193 |
| 9894 | TELO2 | Q9Y4R8 | 0,47112 |
| 54499 | TMCO1 | Q9UM00 | 0,46939 |
| 337867 | UBAC2 | Q8NBM4 | 0,46451 |
| 387990 | TOMM20L | Q6UXN7 | 0,38471 |
| 3300 | DNAJB2 | P25686 | 0,38212 |
| 57154 | SMURF1 | Q9HCE7 | 0,38154 |
| 91942 | NDUFAF2 | Q8N183 | 0,36474 |
| 55742 | PARVA | Q9NVD7 | 0,34263 |
| 83892 | KCTD10 | Q9H3F6 | 0,34099 |
| 90550 | MCU | Q8NE86 | 0,32762 |
| 56159 | TEX11 | Q8IYF3 | 0,28826 |
| 119559 | SFXN4 | Q6P4A7 | 0,27950 |
| 373863 | DND1 | Q8IYX4 | 0,26470 |
| 54708 | MARCHF5 | Q9NX47 | 0,25864 |
| 79888 | LPCAT1 | Q8NF37 | 0,25838 |
| 9463 | PICK1 | Q9NRD5 | 0,25605 |
| 7316 | UBC | P0CG48 | 0,25200 |
| 100287932 | TIMM23 | O14925 | 0,25137 |
| 80262 | PHAF1 | Q9BSU1 | 0,24344 |
| 3105 | HLA-A | P04439 | 0,24250 |
| 267 | AMFR | Q9UKV5 | 0,23924 |
| 10892 | MALT1 | Q9UDY8 | 0,22836 |
| 117584 | RFFL | Q8WZ73 | 0,22656 |
| 5886 | RD23A_HsUbiquit | P54725 | 0,22600 |
| 7189 | TRAF6 | Q9Y4K3 | 0,22263 |
| 1026 | CDKN1A | P38936 | 0,21905 |
| 6383 | SDC2 | P34741 | 0,21422 |
| 8850 | KAT2B | Q92831 | 0,21272 |
| 6566 | SLC16A1 | P53985 | 0,20623 |
| 84447 | SYVN1 | Q86TM6 | 0,20389 |
| 23295 | MGRN1 | O60291 | 0,19926 |
| 83932 | SPRTN | Q9H040 | 0,19307 |
| 29979 | UBQL1 | Q9UMX0 | 0,18722 |
| 5371 | PML | P29590 | 0,18101 |
| 9274 | BCL7C | Q8WUZ0 | 0,17372 |
| 84675 | TRIM55 | Q9BYV6 | 0,17274 |
| 1385 | CREB1 | P16220 | 0,16164 |
| 8878 | SQSTM1 | Q13501 | 0,15212 |
| 4303 | FOXO4 | P98177 | 0,15111 |
| 64219 | PJA1 | Q8NG27 | 0,14827 |
| 9001 | HAP1 | P54257 | 0,14826 |
| 9531 | BAG3 | O95817 | 0,13696 |
| 2130 | EWSR1 | Q01844 | 0,13555 |
| 121536 | AEBP2 | Q6ZN18 | 0,12997 |
| 27146 | FAM184B | Q9ULE4 | 0,11529 |
