# Supplementary material for: The Josephin domain (JD) containing proteins are predicted to bind to the same interactors: Implications for spinocerebellar ataxia type 3 (SCA3) studies using Drosophila melanogaster mutants
Source: Front Mol Neurosci. 2023 Mar 15;16:1140719. doi: 10.3389/fnmol.2023.1140719 (PMC10050893; doi:10.3389/fnmol.2023.1140719)
Supplement: Supplementary file 10 [file Data_Sheet_1.docx]

Supplementary Material

The Josephin domain (JD) containing proteins are predicted to bind to the same interactors: implications for spinocerebellar ataxia type 3 (SCA3) studies using *Drosophila melanogaste*r mutants

Rita Sousa e Silva, André D. Sousa, Jorge Vieira, Cristina P. Vieira

**Supplementary Table 1.** Paralogous human genes of the fly modifiers ATXN3 genes identified by Vobfeldt *et al.* (Vobfeldt *et al*., 2012), according to DIOPT. Stars indicate the genes assigned as suppressors, cardinal those assigned as enhancers, and plus those that are deleterious in flies. In brackets are the original gene names according to Flybase**.**

**Supplementary Table 2.** Paralogous human genes of the fly modifiers ATXN3 genes identified by Zhang *et al*. (Zhang *et al.*, 2010), according to DIOPT.

**Supplementary Table 3.** Paralogous human genes of the fly modifiers ATXN3 genes identified by Bilen and Bonine (Bilen and Bonini, 2007), according to DIOPT. Stars indicate genes are also reported in Vobfeldt et al. (Vobfeldt *et al*., 2012); # indicate genes also reported in Zhang *et al*. (Zhang et al., 2010); & represent the genes used to search human orthologs.

**Supplementary Table 4.** Human ataxin-3 interacome according to EvoPPI3. Proteins analysed using the in-silico methodology are marked with a +. Those that do not produce results, either because ITASSER size restrictions, CPORT, or HADDOCK error, are marked with a, b, c, respectively.

**Supplementary Table 5.** Paralogous human genes of the L1 fly genes identified in EvoPPI as interactors of *Drosophila* Josephin-like protein (JosL; CG3781; 31560).

**Supplementary Table 6**. Human genes described as encoding proteins that are interactors of ataxin-3 and that are paralogous of fly genes described as human ataxin-3 modifiers. Human genes that are not expressed in human basal ganglia, cerebral cortex, midbrain, thalamus, medulla oblongata and pons, tissues relevant for the SCA3 pathology (Seidel et al., 2012) are marked with a star. Fly genes not expressed in the eye and/or brain are marked with a plus signal.

**Supplementary Table 7.** The Jos1 and Jos2 interactor in EvoPPI. Stars indicate common ataxin-3 interactors. Proteins marked with a plus signal have not been studied because of the *in-silico* limitations.

**Supplementary Table 8**. Proteins that present six or more interactions at polyQ tract in the *in-silico* analyses.

**Supplementary Table 9.** Similarity of the ITASSER and AlphaFold protein structures according to the TM-score (Zhang and Skolnick, 2005)

**Supplementary Figure 1**. *ATXN3* (A) and JOSD (B) linages phylogeny across the animal kindom.

**Supplementary Figure 2**. Percentage of interaction per protein residue at the JosL for predicted interactors (those of the ataxin-3; upper panel, in black) and those that are described as the JosL interactors (lower panel, in grey). Dotted boxes represent regions of interaction where more than 50 percent of the proteins show interaction with JosL. The Josephin domain (JD) is assigned in as a dotted box.

**Supplementary Figure 3**. Gene expression (FPKM) and Pearson (*R*) linear correlation for ataxin-3 (square and solid line), Jos1 (triangles and Dot line), and Jos2 (circles and dash line) in striatum (basal ganglia; in black) and mediodorsal nucleus of thalamus (in gray) tissues, at ages 4, 8 13, 19, 21, 23, 30, 36, 37, and 40 years.

**Supplementary Figure 4**. Amino acid alignment of ataxin-3, Jos1, and Jos2. Interaction sites showing a frequency above 50 are assigned in black (the interactors that in interact most at the JD), in grey (the interactors that interact mostly in the C-terminal region) for ataxin-3, in light green (the interactors described in databases), in dark green (the inferred interactors) for Jos1, and in brown (the interactors described in databases), in purple (the inferred interactors) for Jos2.

**Supplementary Figure 5**. Percentage of interaction per protein residue at the exp ataxin-3 for fly paralogous interactors (upper panel, in black) and human interactors (lower panel, in grey). Dotted boxes represent regions of interaction where more than 50 percent of the proteins show interaction with exp ataxin-3. The Josephin domain (JD) is assigned in as a dotted box, where the NES77 and NES141 regions are marked with white boxes. UIM regions are also assigned with grey boxes. The polyQ region is assigned with //.
