# Supplementary figures and images for: The Josephin domain (JD) containing proteins are predicted to bind to the same interactors: Implications for spinocerebellar ataxia type 3 (SCA3) studies using Drosophila melanogaster mutants
Source: Front Mol Neurosci. 2023 Mar 15;16:1140719. doi: 10.3389/fnmol.2023.1140719 (PMC10050893; doi:10.3389/fnmol.2023.1140719)

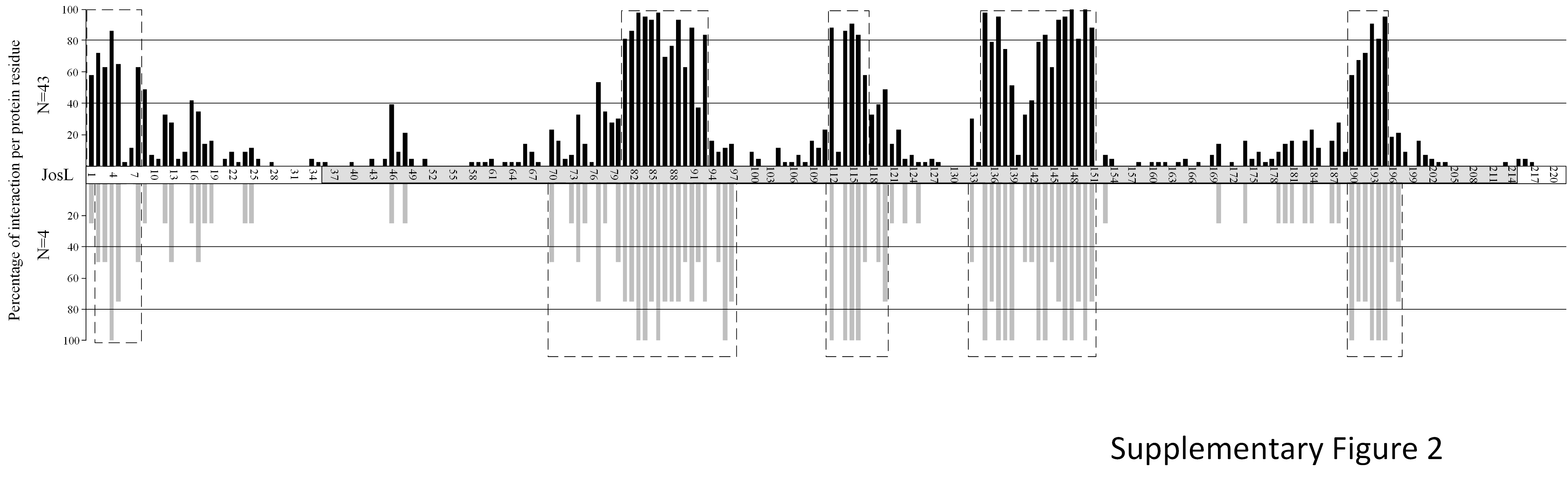

Supplement: Supplementary file 12 [file Image_2.tif]

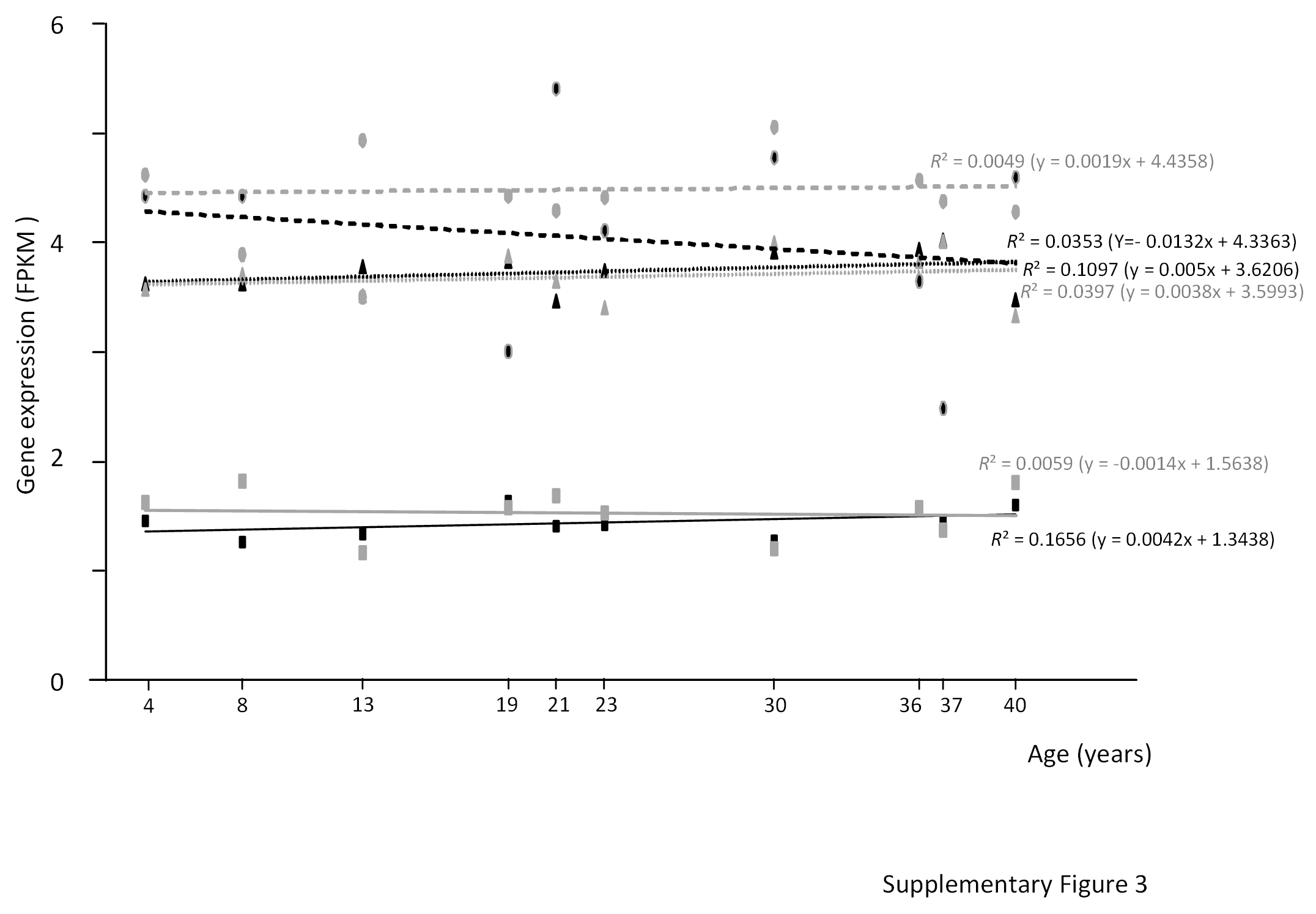

Supplement: Supplementary file 13 [file Image_3.tif]

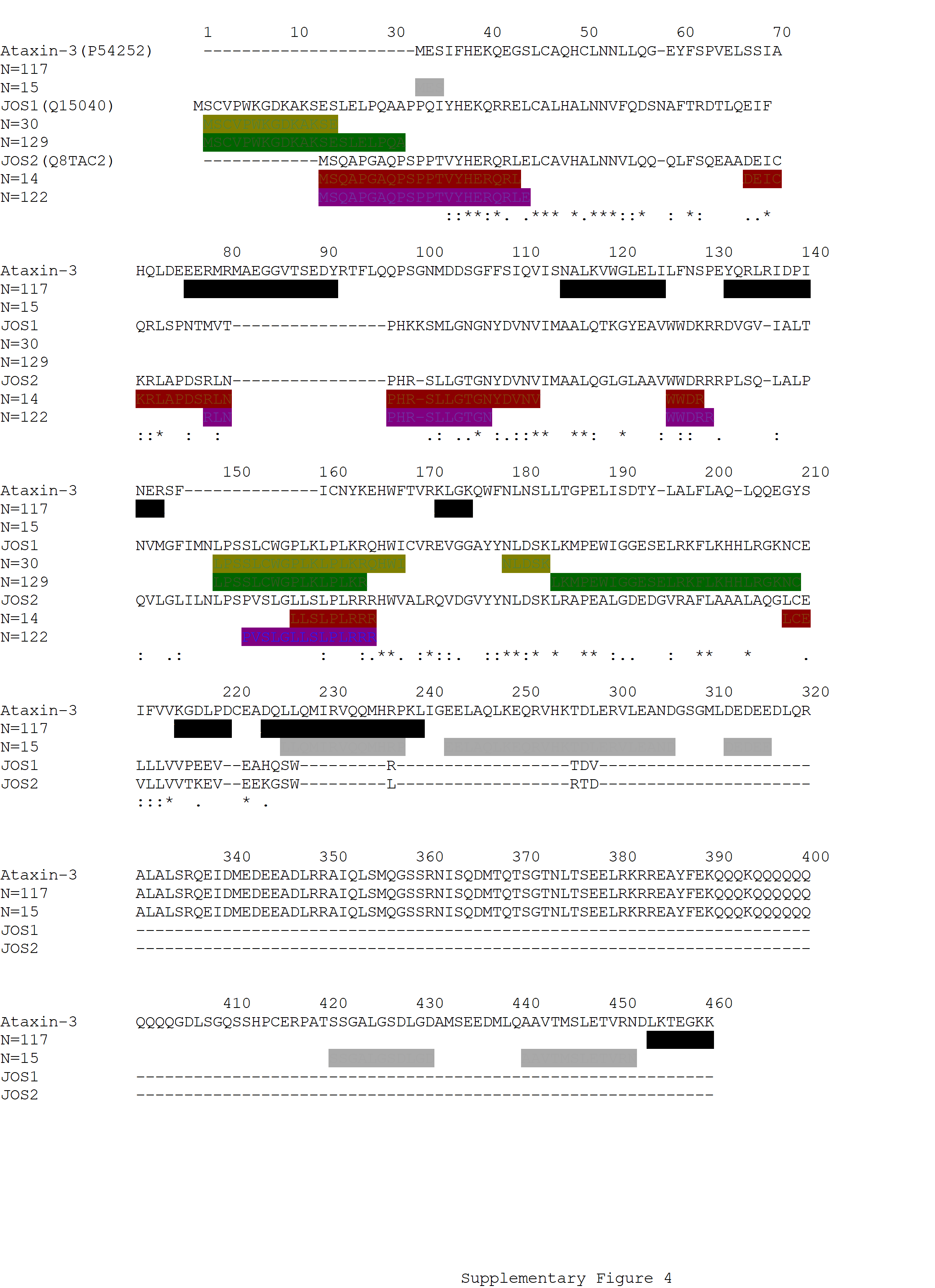

Supplement: Supplementary file 14 [file Image_4.tif]

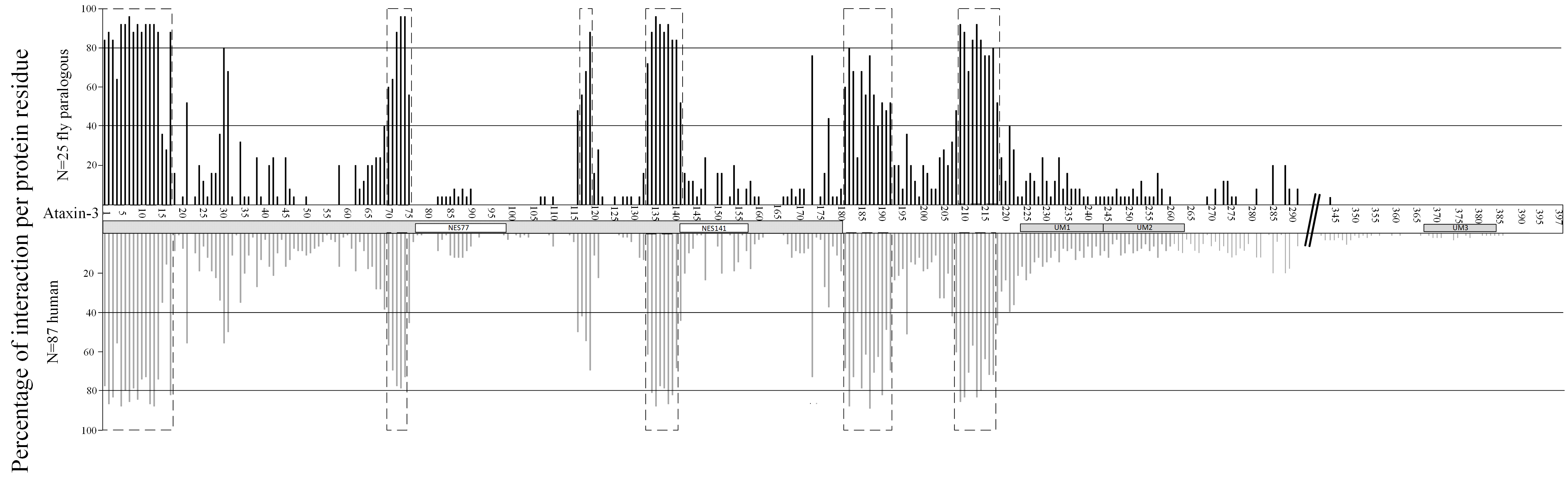

Supplement: Supplementary file 15 [file Image_5.tif]
